# A New Epigenetic Model to Stratify Glioma Patients According to Their Immunosuppressive State

**DOI:** 10.3390/cells10030576

**Published:** 2021-03-05

**Authors:** Maurizio Polano, Emanuele Fabbiani, Eva Adreuzzi, Federica Di Cintio, Luca Bedon, Davide Gentilini, Maurizio Mongiat, Tamara Ius, Mauro Arcicasa, Miran Skrap, Michele Dal Bo, Giuseppe Toffoli

**Affiliations:** 1 Experimental and Clinical Pharmacology Unit, Centro di Riferimento Oncologico di Aviano (CRO) IRCCS, 33081 Aviano, Italy; federica.dicintio@cro.it (F.D.C.); luca.bedon@cro.it (L.B.); mdalbo@cro.it (M.D.B.); gtoffoli@cro.it (G.T.); 2 Department of Electrical, Computer and Biomedical Engineering, University of Pavia, 27100 Pavia, Italy; emanuele.fabbiani01@universitadipavia.it; 3 Centro di Riferimento Oncologico di Aviano (CRO) IRCCS, Division of Molecular Oncology, 33081 Aviano, Italy; eandreuzzi@cro.it (E.A.); mmongiat@cro.it (M.M.); 4 Department of Life Sciences, University of Trieste, 34127 Trieste, Italy; 5 Department of Chemical and Pharmaceutical Sciences, University of Trieste, Via L. Giorgieri 1, 34127 Trieste, Italy; 6 Bioinformatics and Statistical Genomics Unit, Istituto Auxologico Italiano IRCCS, 20095 Cusano Milanino, Italy; davide.gentilini@unipv.it; 7 Department of Brain and Behavioral Sciences, University of Pavia, 27100 Pavia, Italy; 8 Neurosurgery Unit, Department of Neuroscience, Santa Maria della Misericordia University Hospital, 33100 Udine, Italy; tamara.ius@asufc.sanita.fvg.it (T.I.); skrap@asufc.sanita.fvg.it (M.S.); 9 Centro di Riferimento Oncologico di Aviano (CRO) IRCCS, Department of Radiotherapy, 33081 Aviano, Italy; marcicasa@cro.it

**Keywords:** immunosuppression, tumor microenviroment, neural network, genome-wide methylation model, glioma, extracellular matrix

## Abstract

Gliomas are the most common primary neoplasm of the central nervous system. A promising frontier in the definition of glioma prognosis and treatment is represented by epigenetics. Furthermore, in this study, we developed a machine learning classification model based on epigenetic data (CpG probes) to separate patients according to their state of immunosuppression. We considered 573 cases of low-grade glioma (LGG) and glioblastoma (GBM) from The Cancer Genome Atlas (TCGA). First, from gene expression data, we derived a novel binary indicator to flag patients with a favorable immune state. Then, based on previous studies, we selected the genes related to the immune state of tumor microenvironment. After, we improved the selection with a data-driven procedure, based on Boruta. Finally, we tuned, trained, and evaluated both random forest and neural network classifiers on the resulting dataset. We found that a multi-layer perceptron network fed by the 338 probes selected by applying both expert choice and Boruta results in the best performance, achieving an out-of-sample accuracy of 82.8%, a Matthews correlation coefficient of 0.657, and an area under the ROC curve of 0.9. Based on the proposed model, we provided a method to stratify glioma patients according to their epigenomic state.

## 1. Introduction

Gliomas are brain tumors that arise from glial precursor cells. According to their pathological features, gliomas are subdivided in glioblastomas (GBMs), which have the highest grade (IV), and low-grade gliomas (LGGs), a heterogeneous group composed by various tumor types, such as astrocytic, oligodendroglial and ependymal tumors. Gliomas have a heterogeneous clinical outcome with the worse course happening in the GBM group, whereas LGGs are generally less severe. Several biomarkers have been proposed to predict the clinical outcome and response to treatments of gliomas, including genetic and epigenetic ones such as *IDH* mutation and methylation of the *MGMT* promoter. A detailed characterization of glioma-associated molecular signatures has made possible the development of novel therapies, including the use of tyrosine kinase inhibitors. On the other hand, based on the results obtained in the context of other tumors, the use of immune checkpoint inhibitors (ICIs) has been proposed for gliomas, including GBMs. However, despite the recently proposed novel targeted therapy and immunotherapy treatment approaches, treatment strategies for gliomas are in the majority of cases still conventional. In particular, for GBMs, the current standard of care still consists of surgical resection, followed by radiotherapy and chemotherapy [1]. Moreover, so far no immunotherapeutic approach against GBM has demonstrated efficacy in a controlled clinical trial [2,3,4].

The clinical outcome of gliomas is strictly related with the composition and cell cross-talk of tumor microenvironment (TME), in particular with the immune texture in terms of the distinct immune cell types as well as the different immunosuppressive cell populations, such as T regulatory cells (Tregs), myeloid-derived suppressor cells (MDSCs), tumor-associated macrophages (TAMs), dendritic cells and antigen-presenting cells specific to the brain such as microglia [5,6,7]. A significant infiltration of Tregs can be detected in a large fraction of gliomas, in particular in the GBM group. In this context, the activity of *IDO* can contribute to the immunosuppressive state of the TME by creating a tryptophan shortage, which contributes to the suppression of T cell activation and proliferation [8]. Within glioma tumors, microglia and macrophages can represent up to the 12% of the tumor mass [9,10,11,12].

With respect to the macrophages displaying the M1 phenotype, M2 macrophages are more strongly involved in the maintenance of an immunosuppressive state in the TME. Notably, M2 macrophages are generally characterized by the peculiar expression of several cell surface markers including *CD163* [13,14,15,16]. The extracellular matrix (ECM) components such as glycosaminoglycans, glycoproteins, proteoglycans, play a crucial role in the invasion mechanisms of gliomas, mainly through promoting angiogenesis and tumor cell migration. Hypervascularity is a characteristic of gliomas with an increment in angiogenesis compared to healthy brain tissue. This tumor-associated vasculature is not completely formed with leaky vessels and associated with an increase in the interstitial fluid pressure [17].

The degree of immunosuppression of the glioma TME can be associated with a peculiar immunosuppressive signature, with the most accentued immunosuppressive state happening in the case of GBMs [18]. Moreover, specific immunosuppressive features such as depletion of tumor infiltrating lymphocytes (TIL), high PD-L1 expression, and a reduced *IIFN* signature have been associated with recurrent genomic mutations, such as *IDH1*, *TP53*, *NF1*, *PTEN EGFR* and MAPK pathway mutations. Epigenetic modifications including alteration of histone patterns, chromatin structure, changes in microRNA expression levels and DNA methylation status at specific promoters are involved in the modulation of the TME by allowing cells to grow and to escape from immune surveillance. Thus, the immunosuppressive state can be recapitulated by epigenetic regulation, in particular by DNA methylation influencing the expression of transcription factors and regulatory genes related to the immune cell transcriptome. Since DNA methylation plays an important role in cancers, many studies have utilized DNA methylated sequences as biomarkers for cancer detection, including CpG markers and promoter markers. In particular, DNA methylation has been demonstrated to resolve cell of origin of peripheral blood cells [19] and cell-free DNA [20,21,22], and was introduced as a complementary approach to classify central nervous system (CNS) tumors [23]. Moreover, irregular methylations in promoters of cancer-related genes could serve as biomarkers for early cancer diagnosis and prognosis. An example of this is *MGMT* promoter methylation that was demonstrated to be a predictive biomarker for cancer prognosis in GBMs and response to chemotherapy with temozolomide [24,25]. In this context, DNA methylation can be useful to more adequately understand the distribution of the different immune cell subtypes in the context of the TME [26,27,28]. In this study, we fed DNA methylation data into a machine learning model to classify gliomas over their immunosuppression state. We used methylation data as features for our dataset. The target is a novel binary indicator of the immunosuppression state. Due to the limited number of cases available in public datasets, we resorted to both expert and data-driven selection to shrink the number of features and decrease the noise. Given the large number of features and the possibly non-linear nature of the problem, we adopted properly tuned random forest (RF) and deep neural network as classifiers.

We found that the multi-layer perceptron deep outperforms the RF and that a proper feature selection is capable of improving the accuracy of the model. In light of the result of the study, a proper discussion of the biological implications of our study was provided. This classification model could be useful to predict the responsiveness of glioma-affected patients to novel immunotherapeutic approaches, such as the use of ICIs.

## 2. Materials and Methods

### 2.1. Data

The complete workflow, from raw data to the predictive model, is presented in Figure A2.

Our dataset is derived from The Cancer Genome Atlas (TCGA) data hub, available on Xena https://xenabrowser.net (accessed on 12 March 2020). From this source, we extracted the count (FPKM-UQ) of RNA sequencing (RNA-seq) and DNA methylation data for LGG and GBM. The clinical and pathological information of the patients was also gathered from TCGA and the Consortium publication on glioma [29]. The selection of the cases was based on the following criteria: (i) presence of a diagnosed GBM or LGG, (ii) availability of the DNA methylation and RNA sequencing data. A total of 573 cases of brain tumors were enrolled (Table 1).

The input to our machine learning model was made only of methylation data, while the RNA-seq data and the information about the patients were only used in the construction of the target or in ancillary analysis. The methylation at each 5′—C—phosphate—G—3′ (CpG) site is described by the β value, defined as the ratio between the intensity of the methylated probe and the intensity of the total probe. A total of 482,421 CpG sites throughout the genome were assessed and filtered using the procedure described by Bourgon et al. [30], resulting in an initial dataset containing 355,314 CpG probes. We called this dataset AllCpGs.

Taking into account the relevance of M2 macrophages and TReg populations in the modulation of immunosuppression in the context of the TME, cases were labeled for their putative capability of escaping an immunosuppressive state. To do this, we evaluated the immune cells in the TME using *immunedecov* [31]. First, the data relative to RNA-seq were log-transformed and standardized to zero mean and unit variance. We then defined three different criteria based on RNA-seq: (i) expression of the *CD163* gene, (ii) expression of M2 macrophage signature (Macropage M2), and (iii) expression of Tregs signature (T cell regulatory Tregs). The two latter signatures were evaluated using *quantiseq* [32] and *xCell* [33]. The three parameters, (i) to (iii), were used to label cases based on their putative capability of escaping an immunosuppressive state. A case was labelled EvaDe Immune SuppressiON (EDISON) positive if it had more than two out of the three parameters, (i) to (iii), below the first quartile of expression. For the evaluation of the interactions between the immune system and the TME, we leveraged the signatures published on the “Immune-Subtype-Clustering” GitHub repository [34], as proposed in our previous study [35]. The EDISON label was used as a target for our classification models.

### 2.2. Feature Selection

Due to the high number of variables in the DNA methylation data compared to the number of cases, before applying any classification model, we opted to reduce the dimensionality of the input via feature selection. At first, we applied expert selection.

We included in the dataset the CpGs related to the genes which were shown to play crucial roles in gliomas. Specifically, we chose:

1. The genes linked to the putative response of immune suppression in the study by Thorston et al. [18];
2. The genes with the angiomatrix signatures [36];
3. The genes associated with the putative response for ICIs in GBMs [37];
4. The genes reported as with prognostic value for gliomas by Mesrati et al. [38];
5. The genes related to the extracellular matrix (ECM) recently linked to the glioma by Zhao et al. [39].

In order to evaluate the predictive power of different sets of genes, two different datasets were obtained. We called *ImmuneAngioICIs* the one containing the genes described in points 1, 2, and 3, while we called *ImmuneAngioICIsMesECM* the dataset containing the genes described in points 1, 2, 3, 4, and 5.

In order to assess the soundness and effectiveness of our expert selection, we also considered a dataset containing all the CpG probes without any filtering. Our results will show that including all the probes does not result in a better modelling: conversely, the additional features bring noise and worsen the predictive power of our models.

The expert selection reduces the number of CpG in the dataset by a factor of 50. Still, many uninformative features might be present. Given the limited number of available cases, the inclusion of uninformative features results in an increase in the noise and may have detrimental effects on the accuracy of the machine learning model. Therefore, we opted to adopt also a data-driven selection procedure. On each dataset, we applied the Boruta algorithm to detect the set of most relevant features [40]. A scheme with a 10-fold cross-validation and 100 repetitions was adopted. We called *AllCpGs + BORUTA* the dataset resulting after the application of Boruta to *AllCpGs*, *ImmuneAngioICIs + BORUTA* the dataset resulting after the application of Boruta to *ImmuneAngioICIs*, and *ImmuneAngioICIsMesECM + BORUTA* the dataset resulting after the application of Boruta to *ImmuneAngioICIsMesECM*. A summary of the datasets is presented in Table 2.

### 2.3. Modelling

To allow a proper evaluation of the machine learning models, each of the the available datasets, *d*, d∈ {*AllCpGs*, *ImmuneAngioICIs*, *ImmuneAngioICIsMesECM*, *AllCpGs + BORUTA*, *ImmuneAngioICIs + BORUTA*, *ImmuneAngioICIsMesECM + BORUTA*}, was split into a training set $T_{d}$, containing 80% of the samples, and a test set $V_{d}$, including the remaining 20%. The feature selection and the tuning of model hyperparameters were allowed to only take advantage of the training set $T_{d}$, while samples in $V_{d}$ were left apart for the final evaluation. It is important to note that the training sets $T_{d}$ only differ in the inputs, while the target variable and the target sample are the same irrespective of *d*. The same holds for the test sets $V_{d}$. This point is critical to allow for a sound comparison among the performance of the models.

On each dataset, the classification models were then tuned and trained. At first, we considered a RF model. We optimized the hyperparameters, such as the number of trees in the forest, the maximum depth of a tree and the minimum number of samples in a leaf, using a grid-search cross-validation. The tuning procedure followed the one described in Vadalas et al. [41].

On the dataset leading to the best performance metrics, namely *ImmuneAngioICIsMes ECM + BORUTA*, two more models were trained. We selected two architectures of deep neural networks: a multi-layer perceptron (MLP) and a convolutional neural network (CNN). For both models, the hyperparameters such as number of hidden layers, neurons in each layer, and learning rate, were optimized using a grid-search cross-validation.

To further evaluate the complex regulation of methylation effect in different genomic localization, we investigated if the EDISON classification model could be improved by dividing *ImmuneAngioICIsMesECM* and *ImmuneAngioICIs* by regional sites and by applying the RF model.

### 2.4. Evaluation

In addition to the standard accuracy (ACC), we considered the Matthews Correlation Coefficient (MCC), and the area under the receiver operating characteristic (AUC) as performance metrics. First introduced by B.W. Matthews to assess the performance of the prediction of protein secondary structure [42], the MCC has become a widely used measure in biomedical research [43,44]. Due to their large popularity and simple interpretation, MCC and AUC were selected in the US FDA-led initiative MAQC-II, aimed at reaching a consensus on the best practices for the development and validation of predictive models for personalized medicine [43].

The evaluation metrics were computed both in cross-validation, on samples belonging to the train sets $T_{d}$, and on the samples of the test set $V_{d}$. For the cross-validation metrics, the 95% confidence intervals (CIs) were also computed. In order to substantiate the results, the McNemar test was used to assess the significance in performance difference among classifiers [45].

### 2.5. Evaluation of the 338 CpG Probes Used for the Model as Survival Prognosticator

We evaluated the prognostic role of the CpG probes used by the best performing model, i.e., the ones included in *ImmuneAngioICIsMesECM + BORUTA* with survival analysis. In particular, we adopted a random survival forest, an ensemble tree method for the analysis of censored survival data, described by Wang et al. [46]. The hyperparameters of the model were chosen with a randomized search and the feature importance was extracted from the best model using permutation importance.

### 2.6. Definition of a Possible CpG Signature Useful for Liquid Biopsy

The CpG probes used by the best performing model (*ImmuneAngioICIsMesECM + BORUTA*) were also analyzed using the Blood–Brain Epigenetic Concordance (BECon) to assess their possible use in liquid biopsy (https://redgar598.shinyapps.io/BECon/ (accessed on 12 March 2020)). We first chose the CpGs that presented a percentile rank of CpG Change Beta over 75. Then, we applied the least absolute shrinkage and selection operator (LASSO) Cox regression to develop an optimal risk signature with the minimum number of CpGs [47,48]. The correlation of the CpGs with gene expression was also evaluated.

### 2.7. Correlation Analysis between CpGs and Genes

To examine the impact of DNA methylation on the local regulation of gene expression, the Pearson correlation between the β values of the CpGs and the normalized expression of the corresponding genes was calculated. Moreover, in order to investigate the distant regulation of gene expression, we computed the correlation between the β values of CpGs of differentially methylated and expressed genes and the normalized expression of differentially expressed genes.

### 2.8. PPI Network Analysis of DNA Methylation-Driven Genes

The 338 CpG probes used by the best performing model (*ImmuneAngioICIsMesECM + BORUTA*) were mapped by Search Tool for the Retrieval of Interacting Genes (STRING) database (version 10.5 [49] ) by using Cytoscape (3.8.2) [50]. The PPI network was generated based on the medium confidence score of 0.40.

### 2.9. Computational Details

The classification pipeline was built on top of the Scikit Learn library, version 0.20.3 [51] and Python 3.6. All the experiments were run on a 32-core Intel Core i7 workstation with 128GB of RAM running CentOS 7.5. Cox regression and Kaplan–Meier survival curves were computed using R (version 3.6.1) with the survival and survminer packages. The Wilcoxon rank-sum test was used to compare the difference between the groups, while Kruskal–Wallis (K-W) test was adopted to evaluate the differences in risk scores across three or more groups.

## 3. Results

### 3.1. Definition of the EDISON Classification Flag

**Figure 1:**
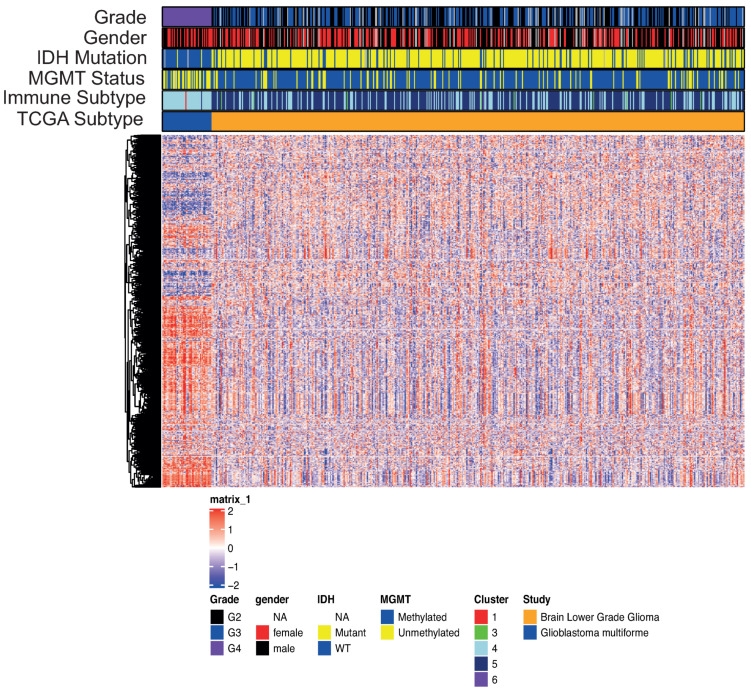
Transcriptomics landscape of patients with either glioblastoma (GBM) or low-grade glioma (LGG). The 2365 genes shown were used to develop the immune cluster subtype by Thorston et al. [18].

We analyzed publicly available datasets of primary glioma samples for which transcriptomic and epigenomic molecular profiles were available. We collected a total of 573 cases, of which 47 cases were GBMs and 506 cases were LGGs. This series of 573 glioma cases was used to develop the model irrespective of being GBMs or LGGs. Figure A2 represents the adopted workflow. Considering the transcriptomics to explore the immune environments landscape (Figure 1), we observed how the different subpopulations of gliomas based on the grade can be described by the the differential expression of some genes, capable of segregating GBMs from LGGs. The LGG group is enriched in *IDH* mutated cases. This is in keeping with previous published results showing that *IDH* mutations are associated with favorable immune composition within the TME and decreased leukocyte chemotaxis, leading to fewer tumor-associated immune cells and better outcome [52]. On the other hand, the GBM group is characterized by a high number of *MGMT* unmetylated cases [24]. Moreover, we evaluated all the cohort for the immune subtype classification, as described in Thorston et al. [18]. With this approach we found that the set of glioma cases employed in the present study is enriched in cases belonging to the subtype 4 (lymphocyte Depleted) and 5 (Immunologically Quiet). These results were in agreement with what previously described showing that the gliomas included in cluster subtype 4 are characterized by a more prominent macrophage signature, with a high M2 response and suppression of the Th1 T cell population, as well as that the glioma cases included in the cluster subtype 5 exhibit the lowest lymphocyte population and the highest macrophage response dominated by M2 macrophages [18,53,54,55].

Based on these characteristics, peculiar of an immune suppressive TME, we chose to assess the immune-related signatures of the 573 sample RNA-seq data by using *immunedecov* (xCell tools) to comprehensively evaluate the transcriptome-based cell-type quantification [31].

**Figure 2:**
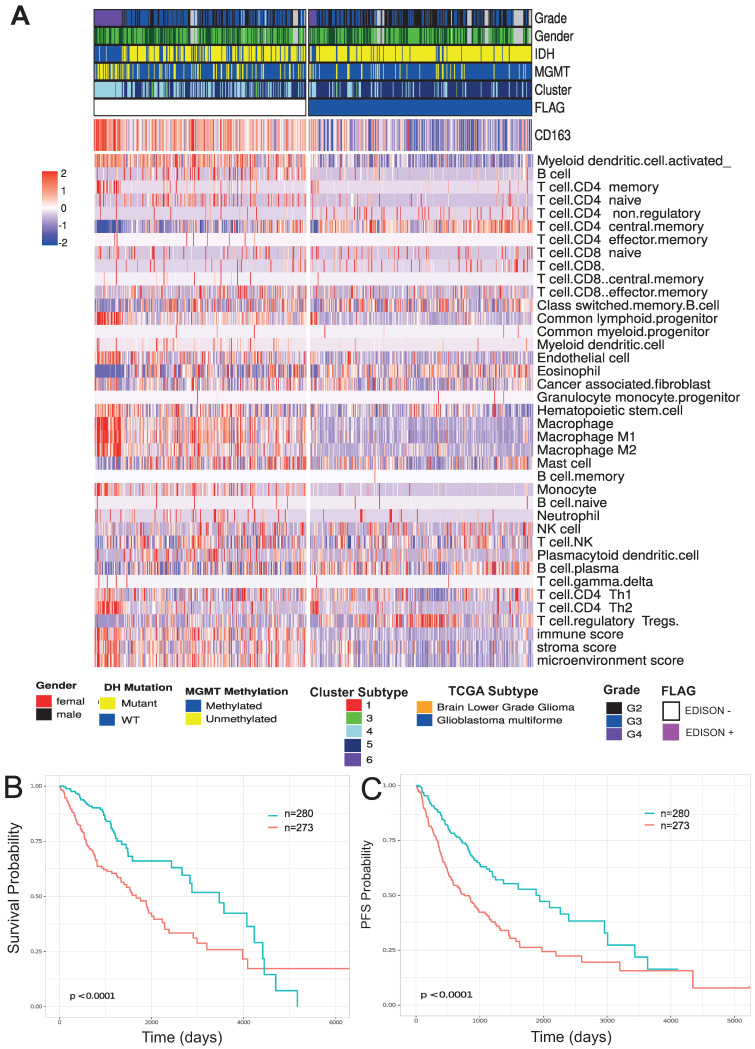
Immune landscape of glioma patients. (**A**) Heatmap of immune signature computed on glioma cohorts from the TCGA study. The signature was calculated using *immunodeconv* (xCell) and the expression of gene CD163. The mutational status and immuno subtype are reported. (**B**) Kaplan–Meier survival curves showing OS interval based on the previously calculated flag on TCGA glioma patients. Time is reported in days. (**C**) Kaplan–Meier survival curves showed progression-free survival (PFS) intervals based on the previously calculated flag on TCGA glioma patients. Time is reported in days.

Figure 2 shows the immune-cell-related gene expression signatures for the glioma cases included in the study. In this context, increasing evidence indicates that TME plays a critical role in supporting the progression of gliomas. In fact, the majority of immune-related cells within brain tumors are macrophages, often comprising up to 30% of the tumor mass [10]. Most TAMs are considered to have M2 phenotype. Increased infiltration of TAMs correlated with improved glioma progression and tumor grade, and predicts poor prognosis in GBM patients. This raises the intriguing possibility that targeting TAMs may be a successful therapeutic strategy for intractable gliomas and GBMs [21]. On the other hand, the capacity to evade the anti-tumoral immune response is associated to the subset of T cells termed *CD4+ CD25+* regulatory T cells (Treg), that have been shown to inhibit the actions of the effector T lymphocytes [5,56]. Thus, we considered the possible influence of two different cell populations, i.e., Tregs and M2 TAMs by evaluating RNA-seq data for gene expression signatures associated with the immunosuppressive role of these two populations. Moreover, we also evaluated the expression of *CD163* itself, being *CD163* one of the most important surface markers of M2 TAMs, that has been recently associated to a prognostic role [14]. We labeled cases as Evade Immune SuppressiON (EDISON) positive with a low immunosuppression state if at least two among these three parameters—*CD163*, M2 TAMs and Tregs—were below the first quartile. The resulting classification describes the possibility that a patient evades the immuno-suppression state and for this reason we called the flag EDISON (EvaDe Immune SuppressiON) positive. Consistently, as reported in Figure 2, EDISON positive cases showed less immunesuppressive phenotypes with both low values of the stromal signature score and the microenviroment signature score, as well as low endothelial signature score [57]. GBM was shown to be characterized by extensive endothelial hyperplasia [58] and the related signatures reported in Figure 2 confirmed this peculiar state.

**Figure A1:**
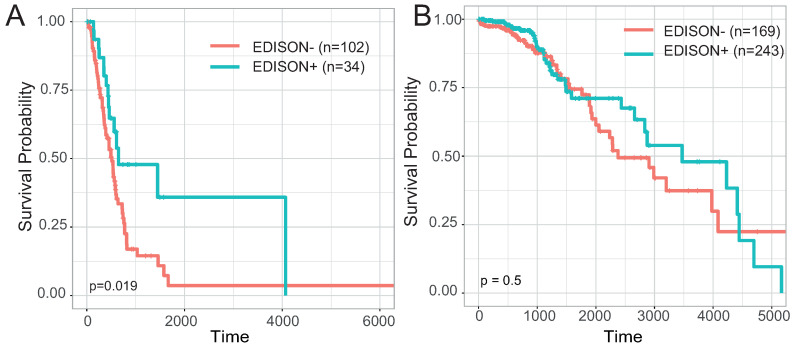

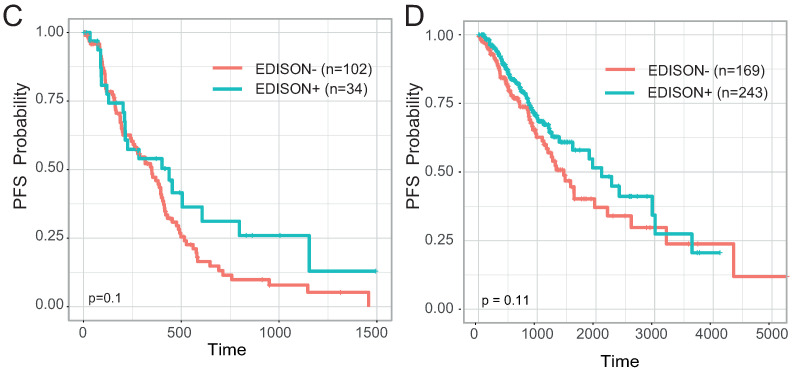
(**A**) Kaplan–Meier survival curves showing OS interval based on the previously calculated flag on TCGA glioma patients with IDH wild-type status. Time is reported in days. (**B**) Kaplan–Meier survival curves showing OS intervals based on the previously calculated flag on TCGA glioma patients with IDH mutated. Time is reported in days. (**C**) Kaplan–Meier survival curves showing PFI interval based on the previously calculated flag on TCGA glioma patients. with IDH wild-type status. Time is reported in days. (**D**) Kaplan–Meier survival curves showing PFS intervals based on the previously calculated flag on TCGA glioma patients with IDH mutated. Time is reported in days.

We also evaluated the capability of the EDISON classification by Kaplan–Meyer for assessing a prognostic significance using both overall survival (OS) and progression-free survival (PFS) intervals. We found that the EDISON positive cases showed significantly longer OS and PFS intervals than EDISON negative cases, thus confirming the importance of the immuno-suppressive-related parameters included in the EDISON flag (Figure 2B,C and Table 3). Figure A1 shows the EDISON classification in the context of IDH mutatant or IDH wildtype cases taken separately for both OS and PFI intervals.

### 3.2. From RNA Genes to the Classification Model

**Figure A2:**
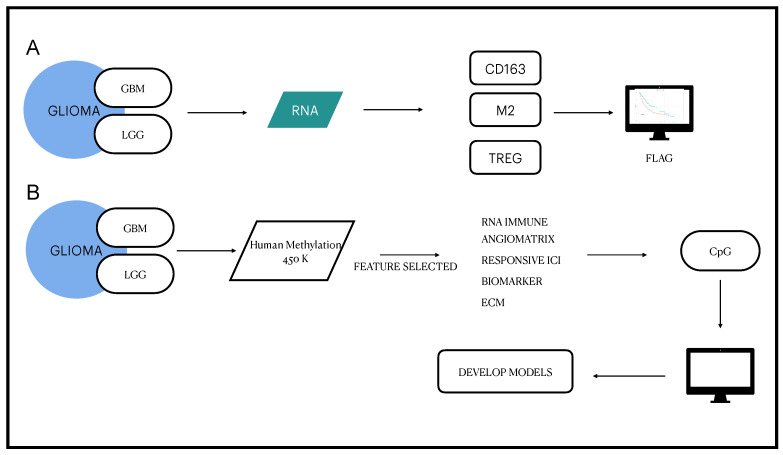
Workflow for the development of a methylation-based machine learning model to predict the immune suppressive state responsiveness of glioma patients. (**A**) Development of the EDISON classification flag using transcriptomic data; (**B**) model construction on methylation dataset (Complete description present in Materials and Methods (Section 2.2).

**Figure A3:**
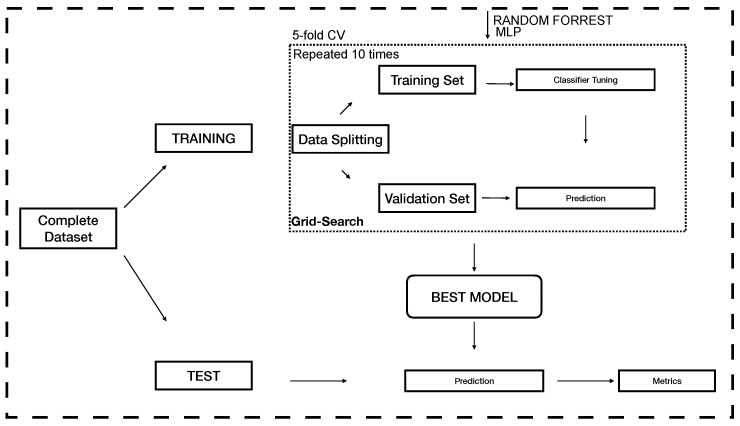
Machine learning workflow for developing the classification model starting by glioma dataset composed by Human Methylation data (450 k) composed by brain low-grade glioma (LGG) patients and glioblastoma (GBM).

The procedure adopted to process the epigenetic data, that includes the creation the EDISON label for the immunosuppressive state, the development of the classification models and their evaluation, is summarized in Figure A2, while a focus on the machine learning models is provided in Figure A3. As described in Section 2.1, we considered a dataset where the input features are β values from CpG probes and the target is a binary label corresponding to the EDISON flag. Starting from the genes used in Thorston et al. [18], we extracted the more informative genes to classify the immunosuppressive state [54,55,59,60,61]. We included also genes associated with the angiogenic signature, according to the prominent role of macrophages in tumor growth and angiogenesis [62], by including the angiomatrix signature reported by Langlois et al. [36]. Moreover, based on the fact that the response of ICIs has been shown to be relevant in both GBM and LGG [63], we evaluated a series of genes putatively related to responsiveness to ICIs, according to the GBM-associated signature reported in Zhao et al. [37]. More precisely, we compared the gene expression of the six GBM cases reported as Responsive against six GBM cases reported as Not Responsive and we obtained that 490 genes were differentially expressed, with adjusted *p*-values lower than 0.01.

The CpG beta values from 450 k Human DNA methylation microarray analysis consisted of 485,577 CpG methylation probes that were pre-processed by applying different basic filters to remove the useless probes, resulting in a final series of 355,314 CpG probes. A total of 6387 CpG probes were included in the overall signature we created and we labeled this set *ImmuneAngioICIs*. On such 6387 CpG probes, a first RF was created (Figure A3), and an out-of-sample MCC of 523 was obtained on the test set V (see Table 4).

Based on a recent review evaluating prognostic genes for GBM [38], we evaluated the possibility of including a second model called *ImmuneAngioICIsMesECM* as described in Section 2.2 [17,39,48]. This procedure created a new set of 6754 CpG probes that were evaluated to classify EDISON positive cases. This second model resulted in an out-of-sample MCC of 0.490.

**Figure 3:**
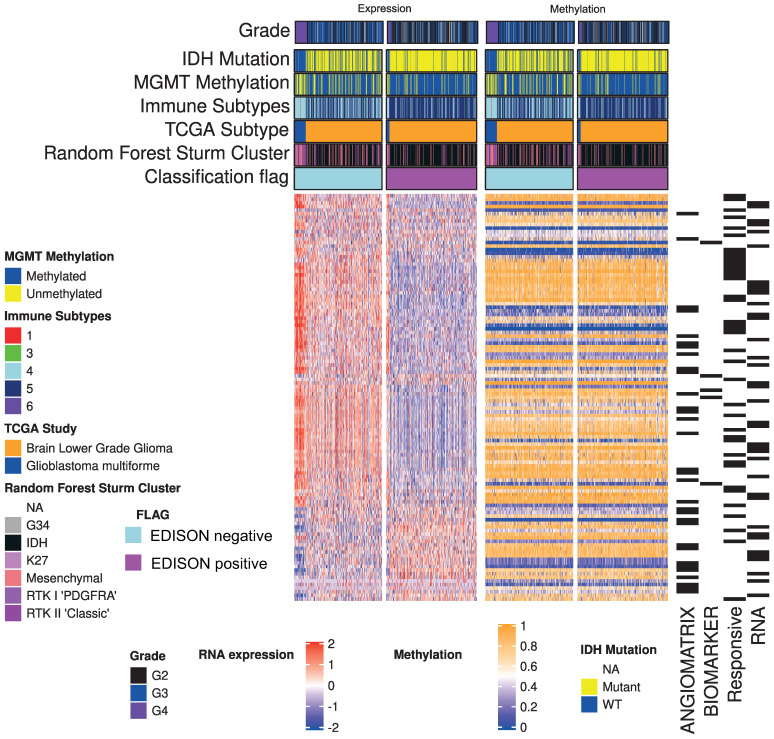
Genome-wide mean methylation status and matched transciptomic landscape from glioma cohort used in this study.

Figure 3 shows the expression of genes included in the model (left panel), and average mean β value for each gene (right panel). While a clearly different expression can be explained for the EDISON classification, the average value for methylation seemed not to be sufficient to capture the methylation status. This result is in agreement with the complex modulation operated by the epigenetic regulation on gene expression. The resulting performance metrics are reported in Table 4. The model trained on *ImmuneAngioICIsMesECM* achieved a better out-of-sample accuracy, but a worse MCC.

The application of a further step of feature selection, with the adoption of Boruta, resulted in an improvement of the metrics achieved by the RF classifiers, with the best results achieved with the dataset *ImmuneAngioICIsMesECM + BORUTA*. The 338 CpGs are listed in Table A2. As reported in Table 4, by using these features selected by Boruta in the datasets *ImmuneAngioICIs + BORUTA* and *ImmuneAngioICIsMesECM + BORUTA*, we obtained an out-of-sample MCC on $V_{ImmuneAngioICIs+BORUTA}$ and $V_{ImmuneAngioICIsMesECM+BORUTA}$ of 0.469 and 0.589, respectively.

Moreover, we evaluated the model fed by all the CpGs, either with or without the adoption of Boruta, and we observed a deterioration in the metrics with respect to our best performing model, trained on *ImmuneAngioICIsMesECM + BORUTA* (Table 4). This evidence substantiates the validity and the effectiveness of the expert selection.

**Figure 4:**
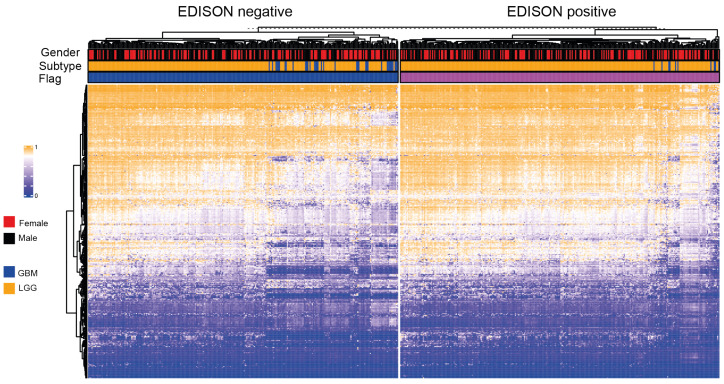
Genomic landscape of the 338 CpG probe selected for the classification model according to the EDISON classification flag.

To further improve the model, we also considered the regional studies of the principal genomic localization such as CpG islands, shores, shelves and open sea. However, by this approach, no improvement in performance was obtained (Table A1). However, shore regions showed a better predictive power with respect to the other regions. This is consistent with previous studies which showed that these regions are more correlated with the regulation of gene expression. Figure 4 shows the genome-wide methylation landscape based on the selected 338 CpG probes, divided by the EDISON flag. Several differences in methylation can be appreciated between EDISON negative and EDISON positive cases. Moreover, in both EDISON positive and EDISON negative categories, GBM and LGG show different behaviours.

### 3.3. Deep Learning for the EDISON Classification

We evaluated the adoption of a deep learning model in place of the RF. Fixing the dataset to *ImmuneAngioICIsMesECM + BORUTA*, we tested both a feed-forward multilayer perceptron (MLP) and a 1D convolutional architecture. We observed better results with an MLP consisting of the input layer (338 neurons), two hidden layers (128 neuron each) and the output layer (1 neuron). Such MLP achieved an out-of-sample MCC of 0.658 and an accuracy of 0.828 on the test set $V_{ImmuneAngioICIsMesECM+BORUTA}$ (Table 5), outperforming the RF model.

**Figure 5:**
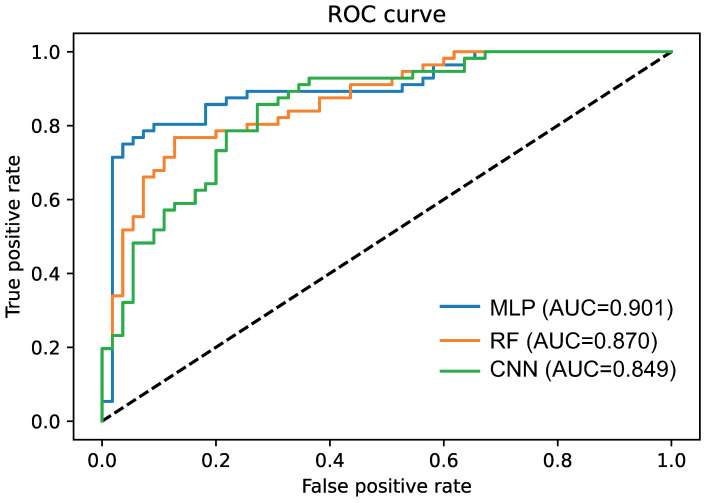
ROC curves of 3 models for EDISON classification using multilayer perceptron (MLP), convolutional neural network (CNN) and random forest (RF). All the models were trained on the dataset *ImmuneAngioICIsMesECM + BORUTA*. The out-of-sample AUC calculated on the test is also reported.

To assess the significance of the difference, we applied the McNemar test. We found that the difference in performance is significant, with a p value of 0.00952. This fact can also be visually appreciated by comparing the ROC curves (Figure 5).

### 3.4. Biological Significance of the Selected CpG Probes

**Figure A5:**
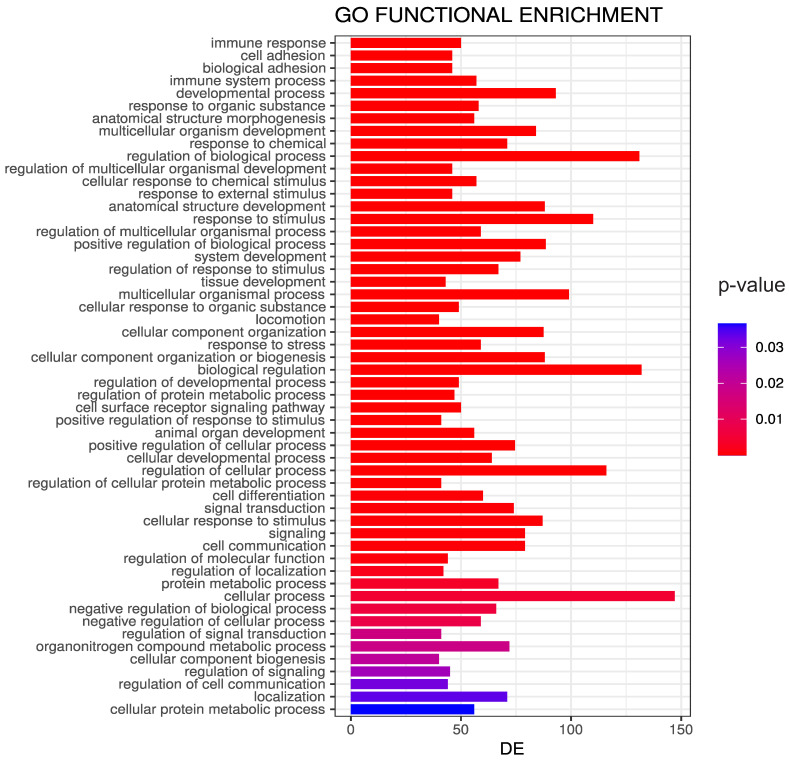
Enrichment of GO terms from 338 CpG probes obtained from the best model. GO terms are plotted according to adjusted *p*-values (BH). Bar sizes represent the number of CpGs translated as genes that fall within a GO category; DE and colour represent the adjusted *p*-values (BH).

To gain insight into the biological significance of the model, we verified if the selected CpGs in *ImmuneAngioICIsMesECM + BORUTA* were correlated with the phenotype we tried to predict by our models. To do so, we applied the g-profile tool [64] to search for an enrichment in GO terms associated with the 338 CpG probes translated in genes. As expected, the selected go-terms were mainly associated with ECM organization, immune response, and regulation of cell adhesion (see Table 6 and Figure A5).

**Figure A6:**
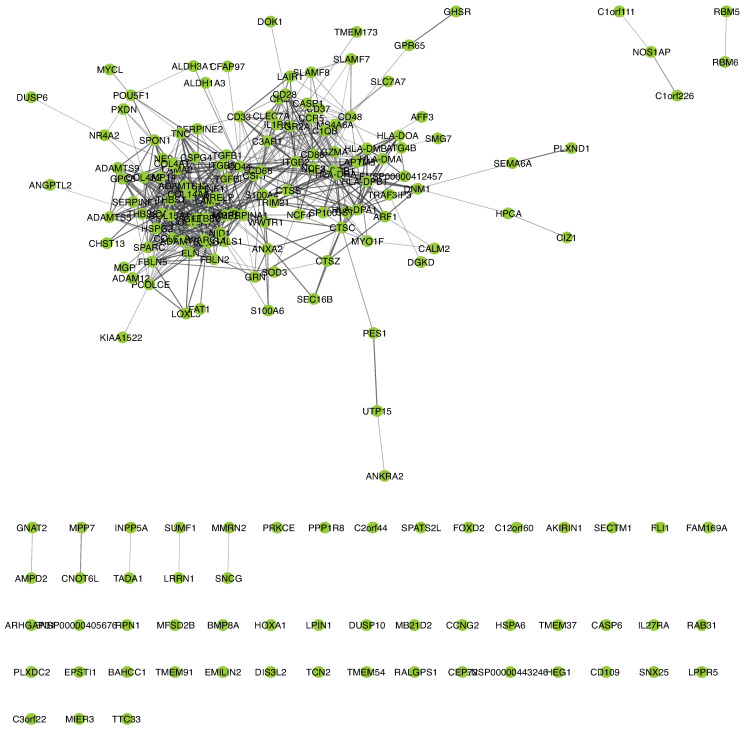
Protein–protein interaction (PPI) network of the genes from 338 CpG probes of *ImmuneAngioICIsMesECM + BORUTA* using STRING in the Cytoscape app [50].

**Figure A7:**
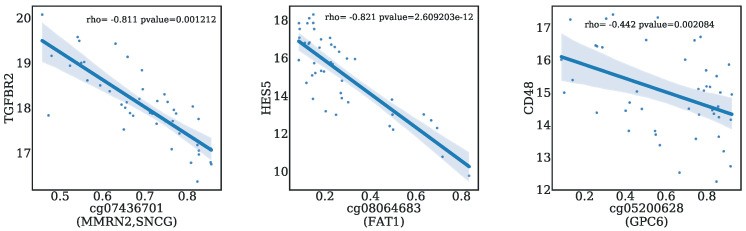

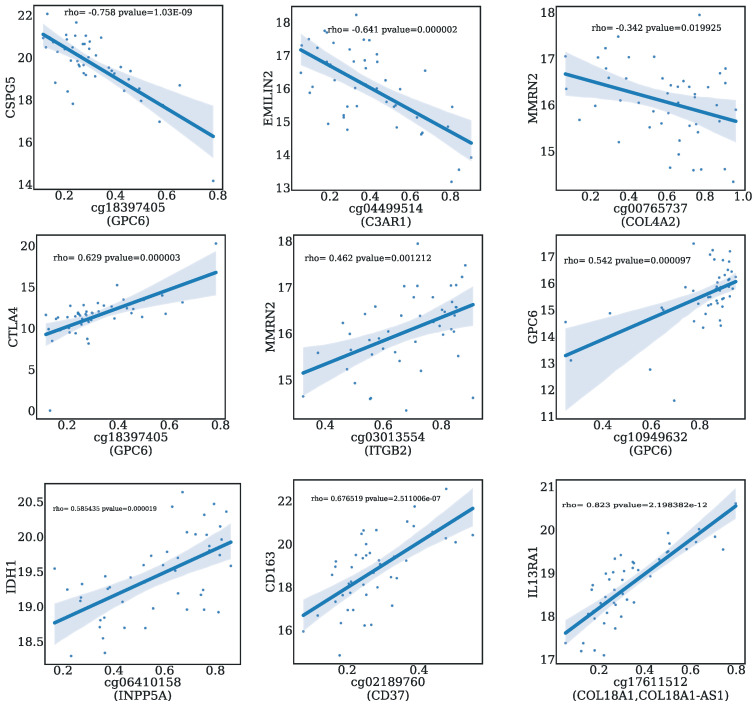
Correlation between CpG probes selected among 338 CpGs with gene expression of some revelant genes. On the x-axis, the methylation status is reported, on the y-axis, RNA expression values are reported. Correlation values by Pearson and *p*-value are reported for each panel.

Moreover, we performed an analysis of the genes related to the 338 CpG probes of *ImmuneAngioICIsMesECM + BORUTA* using STRING in the Cytoscape app (Figure A6). We found that the genes resulted in a linked network of protein–protein interaction (PPI) of 165 nodes and 4058 edges (Figure A5). We also evaluated the involvement of CpG methylation genes in the modulation of the gene expression of gliomas. In Table A8, the CpG probes highly correlated with gene expression are reported. Among these CpGs, we found correlation with genes belonging to angiogenesys pathway, ECM organization, immune response and checkpoint molecules. In Figure A7, several examples of positive and negative correlation are shown.

**Figure 6:**
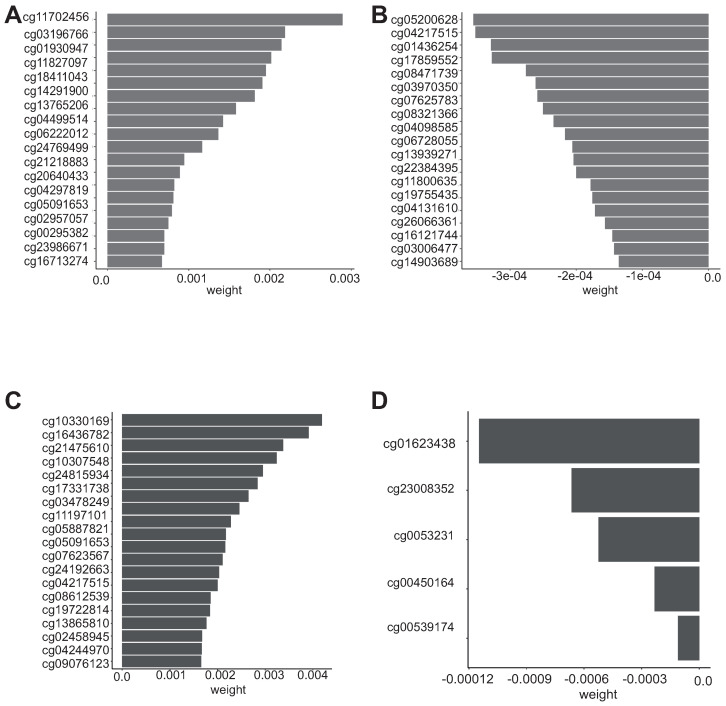
Variable importance of random survival forest model. (**A**) Top 20 CpG probes are reported with positive value influencing the OS interval, (**B**) Top 20 CpG probes are reported with negative influence OS interval, (**C**) Top 20 CpG probes are reported with positive value influencing the progression free survival, (**D**) Top 5 probes are reported with positive value influencing the progression-free survival.

To perform a further selection of the most important CpG among the 338 in *ImmuneAngioICIsMesECM + BORUTA*, we applied random survival forest. The importance values obtained by the permutation analysis are depicted in Figure 6, while overall survival and progression free intervals are reported in Table A3 and Table A4, respectively.

### 3.5. Evaluation of the Transferability of the CpG Methylation Signature in Liquid Biopsy Samples

The methylation signature discussed in this study was obtained from primary glioma samples. However, although DNA methylation is tissue-specific, surrogate tissues such as blood are necessary due to the inaccessibility of human brain samples. Thus, we evaluated the possibility to obtain the genome-wide methylation using the blood to implement a liquid biopsy approach.

**Figure A8:**
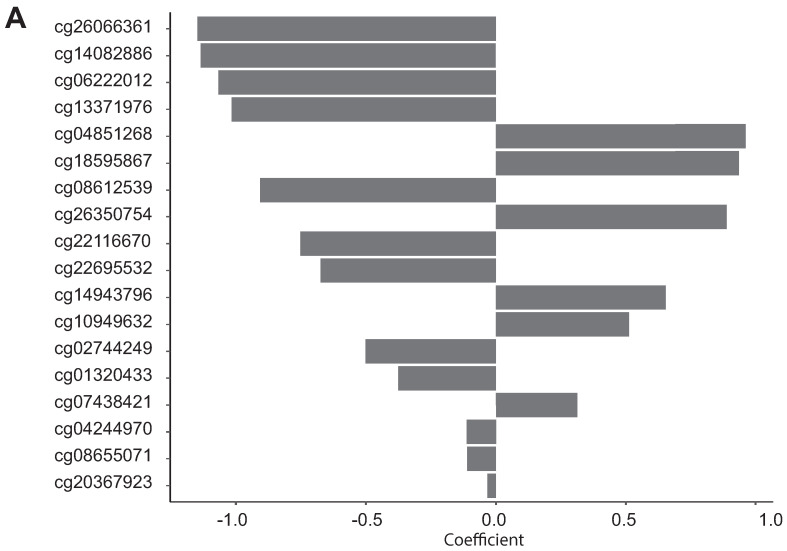

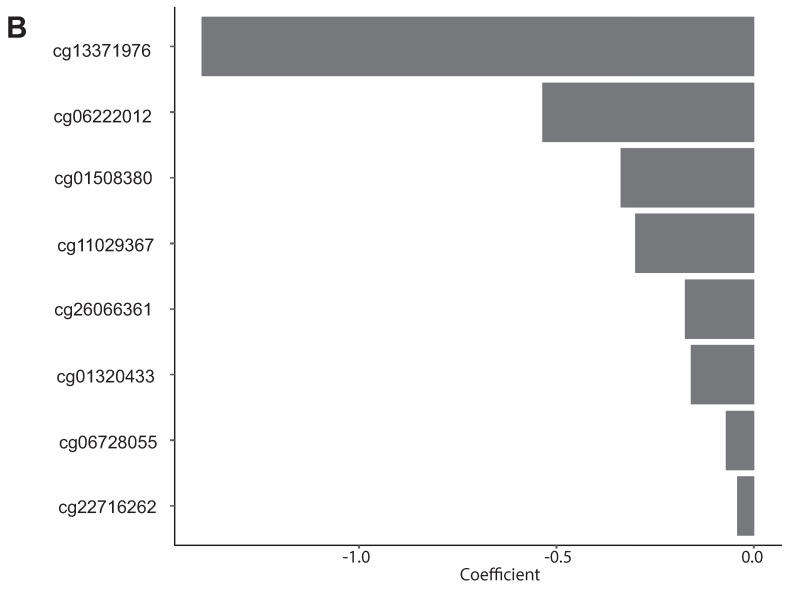
Feature selection using LASSO COXNET of the 113 CpGs selected by BECon. The 18 CpGs selected for OS (**A**) and 8 for PFS interval (**B**) are reported.

BECon (Blood–Brain Epigenetic Concordance; https://redgar598.shinyapps.io/BECon/ (accessed on 12 March 2020) is a tool that allows one to evaluate the concordance of CpGs between blood and brain, and to estimate how strongly a CpG is affected by the cell composition in both blood and brain. To perform such analyses, we imported the 338 CpGs of *ImmuneAngioICIsMesECM + BORUTA* on the BECon software tool and we selected the CpGs which varied in the most consistent way in the blood and in the brain. BECon select 113 CpG probes among 338. A LASSO Coxnet feature selection was then performed to detect the CpGs that can best explained both the overall survival (Figure A8, panel A) and the progression-free interval (Figure A8, panel B). Eighteen CpG probes were selected for the OS interval and eight for PFS (Table A6). The coefficients obtained from LASSO Coxnet were reported in Table A6. Positive values of coefficients were considered risk-associated in contrast to negative values which considered protective-associated. The GO terms analysis performed in positive and negative CpG associated probes is reported in Table A5.

## 4. Discussion

Gliomas are among the most common and aggressive primary tumors in adults [65]. Despite improved insight into the underlying molecular mechanisms, they are still hard to be treated and the prognosis of patients remains poor due to fast progress and scarcity of effective treatment strategies. The highly heterogeneous TME plays a substantial role in tumor malignancy and treatment responses. It is also related to the resistance of glioma cells to chemotherapy [10,59,66,67]. The glioma TME exerts a key role in tumor progression, in particular by providing an immunosuppressive state, with low number of TILs and of other immune effectors cell types as well as a high number of M2 macrophages, that contribute to tumor proliferation and growth [68]. Among the different processes regulating immune escape, TME-associated soluble factors, and/or cell surface-bound molecules are mostly responsible for dysfunctional activity of tumor-specific CD8+T cells. This TME immunosuppression could be involved in the capability of gliomas to respond to ICI treatment. A good understanding of TME and its mutual effects with tumor is important to reveal the treatment resistance mechanisms but also provide new strategies to improve the efficacy of these treatments including immunotherapies [61,69,70,71]. In this study, we systematically evaluated the possibility of creating an epigenetic model to stratify patients according to their capability to evade the immunosuppressive state peculiar of gliomas. We proposed the novel EDISON (EvaDe Immune SuppressiON) flag to summarize the contribution of macrophage M2 and Tregs in the immune suppressive state of gliomas. By comparing a random forest and two different neural network classifiers we showed the superiority of a multi-layer perceptron composed by two hidden layers. Such result is in agreement with that reported by other recent studies [72]. For most of the considered datasets and the models, we recorded higher metrics in the out-of-sample evaluation on the test set with respect to the cross-validation on the train set. This is a symptom of underfitting in the models. The most obvious and effective way to solve the issue, would be to include more samples in the dataset. Unfortunately, we were not able to find larger datasets to integrate our analysis. This could be considered as a limitation even if in an attempt to address the lack of an independent validation set, we followed the recommendations described in Shi et al. [43]. Moreover, further experiments are needed.

The proposed model could be used to predict the capability of the glioma patients to respond to immunotherapy such as ICIs. In this context, the employment of DNA methylation in place of RNA-seq data seems to provide a faster and more cost-effective approach.

Based on the results of the modelling, we defined a set of CpGs to be used as features: we proposed a final series of 338 CpGs related to genes belonging to ECM organization, immune response, angiogenesis and regulation of cell adhesion. Notably, the model trained on the 338 CpGs of *ImmuneAngioICIsMesECM + BORUTA* achieved better out-of-sample metrics than the ones trained on *AllCpGs* and *AllCpGs + BORUTA*. This evidence substantiates the validity and the effectiveness of the expert selection. Finally, we proposed a methylation signature that could be useful in the prediction of the clinical outcome of gliomas when liquid biopsy samples are used. Liquid biopsy represents a minimally invasive procedure that can provide similar information to what is usually obtained from a tissue biopsy samples. We found a small set of CpG (18 CpGs belong OS C and 8 CpGs PFS) that could be easily transferable to the laboratory routine for the classification of glioma patient by using BECon, a tool for interpreting DNA methylation features from blood. This could be useful in the management of glioma patients during the treatments. Moreover, several further suggestions could be highlighted regarding the involvement of the epigenetic modulation of the genes defined by the proposed model in key processes and mechanisms affecting the glioma pathogenesis and progression, such as ECM organization, immune response, angiogenesis and regulation of cell adhesion.

## 5. Conclusions

Despite the advances of molecular understanding and therapies that can be used for glioma treatment, clinical benefits have remained limited. A revelant role in treatment response is exerted by the TME in which the number of TILs and M2 macrophages is responsible for the degree of immunosuppression. In the present study, we proposed an epigenetic model to stratify patients according to their capability to evade the immune suppressive state called EDISON (EvaDe Immune SuppressiON) peculiar of gliomas. We demonstrated the superiority of the neural network composed by two hidden layers to classify the immunosuppressive state with respect to the random forest and convolutional approach. We also proposed a methylation signature that could be useful in the prediction of the clinical outcome of gliomas when liquid biopsy samples are used.

## Figures and Tables

**Table 1:** Cases included in the study from The Cancer Genome Atlas (TCGA) cohorts for Glioma cancer types.

Cohort	Cancer Type	Cases	Cases Flagged as EDISON Positive
LGG	Brain lower grade glioma	506	271
GBM	Glioblastoma multiforme	47	10

**Table 2:** Summary of the datasets, with the number of CpGs included in each one.

Dataset	CpG Count
AllCpGs	355,314
ImmuneAngioICIs	6368
ImmuneAngioICIsMesECM	6754
AllCpGs + BORUTA	3554
ImmuneAngioICIs + BORUTA	512
ImmuneAngioICIsMesECM + BORUTA	338

**Table 3:** Univariate Cox regression analysis of OS and PFS in the entire cohort included in the study using classification derived from RNA-seq data.

Endpoint	Status	Number of Samples	HR	95% CI for HR	*p* Value
OS	EDISON+	n = 553	0.55	0.39–0.77	<0.01
PFI	EDISON+	n = 553	0.57	0.43–0.75	<0.01

Abbreviations: OS, overall survival; PFI, progression-free survival; HR, hazard ratio; CI, confidence interval.

**Table 4:** Metrics obtained for the random forest model on different datasets. The metrics were computed both in cross-validation (CV) on the train set T (mean with 95% confidence intervals) and in out-of-sample evaluation on the test set V. In **bold**, the best performer.

Dataset	ACC CV (CI)	ACC Test	MCC CV (CI)	MCC Test
AllCpGs	0.713 (0.676–0.747)	0.756	0.435 (0.359–0.502)	0.538
ImmuneAngioICIs	0.7155 (0.679–0.754)	0.716	0.436 (0.368–0.512)	0.523
ImmuneAngioICIsMesECM	0.710 (0.674–0.748)	0.739	0.429 (0.354–0.504)	0.490
AllCpGs + BORUTA	0.736 (0.699–0.770)	0.755	0.478 (0.404–0.547)	0.532
ImmuneAngioICIs + BORUTA	0.717 (0.681–0.752)	0.729	0.443 (0.373–0.511)	0.469
**ImmuneAngioICIsMesECM + BORUTA**	**0.747 (0.713–0.780)**	**0.793**	**0.498 (0.432–0.563)**	**0.589**

**Table 5:** Metrics obtained for the random forest and the MLP model on dataset *ImmuneAngioICIsMesECM + BORUTA*. The metrics were computed both in cross-validation (CV) on the train set T (mean with 95% confidence intervals) and in out-of-sample evaluation on the test set V. In **bold**, the best performer.

Model	ACC CV (CI)	ACC Test	MCC CV (CI)	MCC Test
RF	0.747 (0.713–0.780)	0.793	0.498 (0.432–0.563)	0.589
**MLP**	**0.807 (0.795–0.819)**	**0.828**	**0.625 (0.601–0.647)**	**0.657**

**Table 6:** Top 30 terms’ signatures from enrichment analysis using gProfile on 338 CpG probe from the best model [64].

#Term ID	Term Description	Observed Gene Count	Background Gene Count	Strength	False Discovery Rate
GO:0030198	extracellular matrix organization	31	296	1.14	$1.01\times 10^{-21}$
GO:0006955	immune response	43	1560	0.56	$1.14\times 10^{-10}$
GO:0002376	immune system process	49	2370	0.43	$3.46\times 10^{-8}$
GO:0030155	regulation of cell adhesion	23	623	0.68	$4.40\times 10^{-7}$
GO:0048514	blood vessel morphogenesis	18	381	0.79	$7.85\times 10^{-7}$
GO:0001568	blood vessel development	19	464	0.73	$2.19\times 10^{-6}$
GO:0007155	cell adhesion	25	843	0.59	$3.49\times 10^{-6}$
GO:0009653	anatomical structure morphogenesis	40	1992	0.42	$3.56\times 10^{-6}$
GO:0001525	angiogenesis	15	297	0.82	$3.73\times 10^{-6}$
GO:0035239	tube morphogenesis	21	615	0.65	$3.73\times 10^{-6}$
GO:0048583	regulation of response to stimulus	59	3882	0.3	$7.44\times 10^{-6}$
GO:0010033	response to organic substance	48	2815	0.35	$8.17\times 10^{-6}$
GO:0035295	tube development	23	793	0.58	$1.03\times 10^{-5}$
GO:0002684	positive regulation of immune system process	24	882	0.55	$1.54\times 10^{-5}$
GO:0071310	cellular response to organic substance	40	2219	0.37	$3.37\times 10^{-5}$
GO:2000026	regulation of multicellular organismal development	36	1876	0.4	$3.54\times 10^{-5}$
GO:0007492	endoderm development	8	76	1.14	$3.63\times 10^{-5}$
GO:0050896	response to stimulus	91	7824	0.18	$3.99\times 10^{-5}$
GO:0050776	regulation of immune response	23	873	0.54	$4.03\times 10^{-5}$
GO:0045765	regulation of angiogenesis	13	277	0.79	$4.46\times 10^{-5}$
GO:0045321	leukocyte activation	23	894	0.53	$5.56\times 10^{-5}$
GO:0002443	leukocyte mediated immunity	19	632	0.59	$6.19\times 10^{-5}$
GO:0070887	cellular response to chemical stimulus	44	2672	0.33	$6.19\times 10^{-5}$
GO:0002274	myeloid leukocyte activation	18	574	0.61	$6.69\times 10^{-5}$
GO:0010757	negative regulation of plasminogen activation	4	6	1.94	$6.84\times 10^{-5}$
GO:0051239	regulation of multicellular organismal process	45	2788	0.32	$6.84\times 10^{-5}$
GO:0002682	regulation of immune system process	29	1391	0.43	$8.66\times 10^{-5}$
GO:0006027	glycosaminoglycan catabolic process	7	62	1.17	$8.66\times 10^{-5}$
GO:0050778	positive regulation of immune response	18	589	0.6	$8.66\times 10^{-5}$

**Figure A4:**
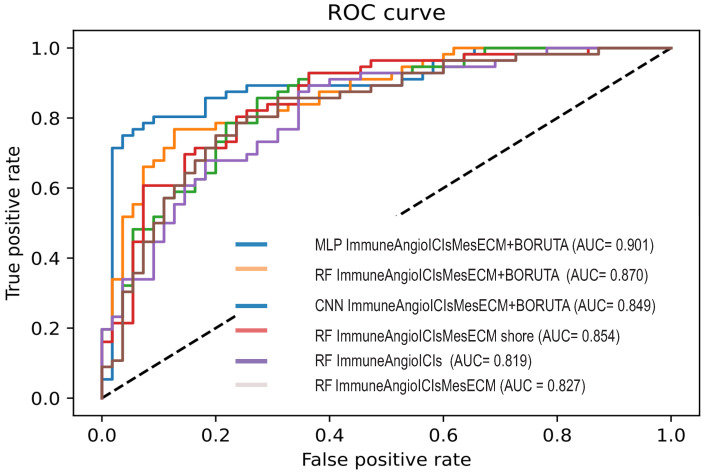
ROC curves of some models for EDISON classification with the indication of dataset used and the machine learning model used. The out-of-sample AUC calculated on the test is also reported. Random Forest (RF); multi-layer perceptron (MLP); convolutional neural network (CNN).

## Data Availability

All the results here showed are based on data generated by the TCGA Research Network: https:/cancer.gov/tcga (accessed on 12 March 2020).

## Abbreviations

The following abbreviations are used in this manuscript:

MDPI	Multidisciplinary Digital Publishing Institute
DOAJ	Directory of open access journals
TLA	Three-letter acronym
LD	Linear dichroism
TME	Tumor microenvironment
EDISON	Evade immunosuppresion
TCGA	The Cancer Genome Atlas
GBM	Glioblastoma
LGG	Low-grade glioma
CI	Confidence interval
MLP	Multi-layer percerptron
RF	Random Forest
CNN	Convolutional neural network
ECM	Extracellular matrix
ICIs	Immune checkpoint inhibitors
BECon	Blood–Brain Epigenetic Concordance
MCC	Matthew correlation coefficient
ACC	Accuracy
Tregs	T regulatory cells
MDSCs	Myeloid-derived suppressor cells
TAMs	Tumor-associated macrophages

## Appendix A

**Table A1:** Model metrics in cross-validation (mean with confidence intervals) and on the test set using CpG probes derived from RNA. ACC: accuracy; MCC: Matthews Correlation, prec: Precision, recal: Recall Coefficient; CI: 95% studentized bootstrap confidence interval; RF: Random Forest.

Model	Regions	ACC (CI)	ACC Test	MCC (CI)	MCC Test
**RF**	IImmuneAngioICIsMesECM-ISLAND	0.724 (0.688–0.769)	0.747	0.460 (0.389–0.549)	0.522
**RF**	ImmuneAngioICIsMesECM-OPENSEA	0.725 (0.689–0.762)	0.691	0.456 (0.386–0.533)	0.469
**RF**	ImmuneAngioICIsMesECM-SHORE	0.749 (0.705–0.790)	0.774	0.501 (0.417–0.583)	0.553
**RF**	ImmuneAngioICIsMesECM-SHELF	0.758 (0.722–0.789)	0.747	0.529 (0.459–0.593)	0.510
**RF**	ImmuneAngioICIs-ISLAND	0.756 (0.716–0.792)	0.758	0.514 (0.439–0.587)	0.518
**RF**	ImmuneAngioICIs-SHORE	0.753 (0.710–0.798)	0.734	0.509 (0.422–0.598)	0.536
**RF**	ImmuneAngioICIs- OPENSEA	0.729 (0.757–0.700)	0.738	0.463 (0.406–0.520)	0.490
**RF**	ImmuneAngioICIs-SHELF	0.725 (0.685–0.763)	0.720	0.457 (0.373–0.537)	0.543

**Table A2:** 338 CpG probes included in best model to classify patient according to the EDISON flag.

CpG	Gene
cg01681098	SENCR, FLI1
cg24457026	GRN
cg13909178	RP11-744N12.3 FLI1
cg03531211	XXbac-BPG181M17.5, HLA-DMA
cg04917472	CTSZ
cg21012874	MMRN2, SNCG
cg13662634	RALGPS1, ANGPTL2
cg17054708	FBLN2
cg10453850	AL645941.1, HLA-DMB, XXbac-BPG181M17.5
cg23008352	COL4A1
cg24421410	XXbac-BPG181M17.5, HLA-DMA
cg07852825	GHSR
cg04499514	C3AR1
cg16436782	RP11-212E4.1, COL4A1
cg03677069	MMRN2, SNCG
cg00215182	C1QB
cg13353679	AFF3, AC092667.2
cg14082886	CD44
cg09552892	MMRN2, SNCG
cg04275881	SLAMF8
cg02072495	ANXA2
cg00338116	EPSTI1
cg10762214	INPP5A
cg10070185	SERPINA1
cg13810673	GPR65
cg07857225	PLXND1
cg11037750	TGFB1
cg07450037	HOTAIRM1, HOXA1, HOTAIRM1_1
cg22568423	MYO1F
cg01436254	CD86
cg17451419	CYR61
cg18273417	S100A4
cg18837947	CCNG2
cg27565899	AMPD2
cg07625783	SLAMF8
cg13371976	PRELP
cg24815934	ITGB2
cg17599241	VCAN-AS1, VCAN
cg10518264	HLA-DMB, XXbac-BPG181M17.5
cg11800635	DOK1, LOXL3
cg26357596	GZMA
cg09456094	SP100
cg11827097	SP100
cg04131610	CCR5, RP11-24F11.2
cg00609834	SPON1
cg08076018	RALGPS1, ANGPTL2
cg06746774	KIAA1522
cg13700051	TTC33
cg17928895	CTSZ
cg15550100	ATG4B
cg07251141	ADAM12
cg26969179	ADAM12
cg18245281	CTSZ
cg00539174	CTSZ
cg17571335	FLI1
cg25428929	ATG4B
cg01536987	EPSTI1
cg20694619	TRAF3IP3
cg03970350	PES1, TCN2
cg13765206	EMILIN2
cg04217515	ITGB2
cg14994258	PXDN
cg11029367	HEG1
cg00765737	COL4A2
cg07464217	CTSZ
cg03075156	PRKCE
cg08655071	TRAF3IP3
cg00295382	MYCL
cg14903689	COL18A1
cg19408145	CD48
cg17420036	HSPG2
cg18274749	HSPG2
cg07436701	MMRN2, SNCG
cg02744249	CTSZ
cg22116670	CTB-113P19.1, SPARC
cg24192663	HSPA6, RP11-25K21.6, FCGR2A
cg13785221	ANXA2
cg17801352	PXDN
cg05887821	INPP5A
cg18411043	LAPTM5
cg03478249	EPSTI1
cg21936552	BAHCC1
cg05200628	CD48
cg01930947	C1orf111, RP11-565P22.6, C1orf226
cg10330169	DIS3L2
cg10587741	LGALS1
cg24539923	SERPINE1
cg10768321	CTC-301O7.4, CD37
cg09538921	IL27RA, CTB-55O6.4
cg18968623	INPP5A
cg08064683	FAT1
cg06330722	PCOLCE, PCOLCE-AS1
cg10307548	SOD3
cg09707038	CALM2, RP11-761B3.1
cg16024530	FLI1
cg13790288	CD28
cg08139855	CSF1
cg19919590	LAPTM5
cg20600379	HLA-DMB, XXbac-BPG181M17.5
cg24375627	S100A6
cg12339920	TGFBI
cg27617132	INPP5A
cg03682712	LOXL1, LOXL1-AS1
cg21746573	PRKCE
cg19506628	CEP72
cg17319576	CYR61
cg17911539	C3orf22, CHST13
cg04232128	TMEM173
cg05360958	C12orf60, MGP
cg04755674	IL27RA, CTB-55O6.4
cg03013554	ITGB2
cg04297819	HSPG2
cg00799121	ADAMTS2
cg08321366	MMP14
cg19722814	SERPINE1
cg14943796	BAHCC1
cg04771838	COL4A2
cg11581627	CD33
cg14991595	MB21D2
cg15347156	MMRN2
cg04153551	FBLN5
cg06222012	AC078941.1, AC023115.2
cg04244970	SLAMF7
cg22704788	PRELP
cg21043746	ADAMTS2
cg26532826	PES1, TCN2
cg13962321	HIST2H2BB, RP5-998N21.7, RP5-998N21.10
cg11702456	SP100
cg09076123	NCF2, SMG7
cg08825225	FLI1
cg17713010	LAIR1
cg15522984	LAMC1
cg08682341	INPP5A
cg03813885	CFAP97, SNX25
cg10845380	SLC7A7
cg12613839	ADAMTS2
cg02588309	TTC33
cg02189760	CTC-301O7.4, CD37
cg16925003	PXDN
cg07947930	PRELP
cg06410158	INPP5A
cg24644113	TADA1
cg27547543	POU5F1
cg21860679	DUSP6, RP11-823E8.3
cg27329371	ALDH3A1
cg00771084	ATG4B
cg11594010	INPP5A
cg11301254	TTC33
cg09926389	TGFB1
cg03982087	RAB31
cg02286081	HLA-DPA1, HLA-DPB1
cg26025068	PPP1R8
cg00078334	MMP2
cg23638686	INPP5A
cg19755435	GPR65
cg08530414	RP4-607I7.1, CD44
cg15999547	TMEM54, HPCA
cg26214645	SECTM1
cg25206536	MIR572
cg20502977	COL6A3
cg23659056	FOXD2, FOXD2-AS1
cg23986671	ADAMTS5
cg26138144	LGALS1
cg07855465	BAHCC1
cg03196766	THBS1
cg17859552	INPP5A
cg18900669	RP11-186B7.4, CD68
cg19915711	EPSTI1
cg10974980	LOXL1
cg08612539	CTA-833B7.2, NCF4
cg18397405	GPC6
cg00450164	TRAF3IP3
cg26650846	ADAMTS2
cg04098585	CD28
cg16826739	INPP5A
cg24767336	TGFB1, CTC-435M10.3, TMEM91
cg03006477	CD109
cg16713274	COL18A1, LL21NC02-21A1.1
cg25450450	CTB-118N6.2, SEMA6A
cg09277376	FOXD2-AS1
cg03440588	FOXD2, FOXD2-AS1
cg24129356	XXbac-BPG181M17.5, HLA-DMA
cg16121744	COL18A1
cg14139008	DNM1
cg24226528	TMEM37
cg11875119	PES1, TCN2
cg01508380	MMP14
cg09280946	CTSC
cg02543462	IL1RN
cg00142150	LGALS1
cg21005525	ARF1
cg07697770	TGFBI
cg03930369	COL4A2
cg06671298	BAHCC1
cg15254671	MYO1F
cg00292662	LGALS1
cg21236655	TNC
cg07724259	EMILIN2
cg23865240	HOTAIRM1, HOXA1
cg09321817	HLA-DPA1
cg18595867	FOXD2-AS1
cg22158252	BMP8A
cg27438456	INPP5A
cg07085815	SERPINE2
cg18644834	ANKRA2, UTP15
cg24287218	HLA-DPA1
cg24707889	ITGB2, ITGB2-AS1
cg09269866	FOXD2, FOXD2-AS1
cg03753191	EPSTI1
cg22716262	MPP7
cg22595235	SUMF1, LRRN1
cg19575208	HLA-DRB1
cg06507307	INPP5A
cg13939271	DNM1
cg23225572	RP11-565P22.6, NOS1AP, C1orf226
cg11197101	KIAA1522
cg21869219	ARHGAP31
cg10954654	CTSS
cg20481110	SECTM1
cg11804789	CST7
cg25214684	AKIRIN1
cg15114672	VCAN
cg00516966	ALDH3A1
cg14791054	RP11-66B24.4, ALDH1A3
cg00816609	FBLN2
cg03055440	MS4A6A
cg21218883	PRKCE
cg02458945	MMP2
cg22118297	ADAMTS9, ADAMTS9-AS1
cg20640433	LAMA2
cg12689670	LAMC1
cg03573861	BAHCC1
cg07438421	SERPINF1
cg05822532	ELN
cg15849060	ALDH3A1
cg02784696	C2orf44, MFSD2B
cg26399819	MIER3
cg18832223	CEP72
cg09777237	ELN
cg15504747	PLXND1
cg01338658	LAMC1
cg00894134	DNM1
cg25306579	INPP5A
cg00532319	RPN1
cg07906179	BAHCC1
cg24493834	LAMA2, MESTP1
cg22136020	CSPG4
cg01320433	XXyac-YX65C7_A.2, THBS2
cg10989879	CFAP97, SNX25
cg15459165	LAPTM5
cg01623438	CTSZ
cg12253414	ITGB5
cg00777079	SERPINF1
cg08638320	FOXD2, FOXD2-AS1
cg05831823	CR2
cg12630520	SPARCL1
cg23446438	MYO1F
cg06728055	WWTR1
cg05492532	INPP5A
cg09545579	BAHCC1
cg26204079	RP11-400N9.1, DGKD
cg14291900	SLC7A7
cg21475610	CCNG2
cg07575373	CTC-301O7.4, CD37
cg05658236	FOXD2-AS1
cg15046675	CTC-301O7.4, CD37
cg22216491	CASP6
cg05091653	SP100
cg11076970	HLA-DOA
cg26262232	XXbac-BPG181M17.5, HLA-DMA
cg25645491	HLA-DRA
cg23173573	DUSP10
cg14880894	CNOT6L
cg02316283	MMP14
cg05041061	BAHCC1
cg12937501	AC106875.1, LPIN1
cg26034531	LPPR5, RP5-896L10.1
cg06390079	ALDH3A1
cg01120369	PLXND1
cg18764513	SLC7A7
cg05830842	COL14A1
cg11728145	PXDN
cg07659054	HOTAIRM1, HOXA1
cg13802966	CASP1
cg13865810	COL15A1, RP11-92C4.6
cg07623567	HLA-DMB, XXbac-BPG181M17.5
cg11912272	SPATS2L
cg17016011	INPP5A
cg00416645	AC007563.5, IGFBP5
cg01997629	TRAF3IP3
cg10928302	RBM6, RBM5
cg02957057	NID1
cg17081489	RP4-798P15.3, SEC16B
cg10001720	LAPTM5
cg20407868	INPP5A
cg24769499	TMEM37
cg26350754	HLA-DPA1, HLA-DPB1
cg10949632	GPC6
cg22905097	EPSTI1
cg26066361	CLEC7A
cg09099927	RP11-333E13.4
cg17611512	COL18A1, COL18A1-AS1
cg13477614	BAHCC1
cg25913233	CTB-113P19.1, SPARC
cg07616471	CCR5, RP11-24F11.2
cg04654716	CTD-2377O17.1, FAM169A
cg08471739	PLXND1
cg27297192	INPP5A
cg04851268	GHSR
cg24931346	C1QB
cg21784272	FAT1
cg22987448	MYO1F
cg22164238	AMPD2, GNAT2
cg08288016	FAT1
cg21398469	CCNG2
cg22384395	RP11-66B24.9, ALDH1A3
cg05710142	KIAA1522
cg21904489	ARHGAP31
cg01975495	SERPINE1
cg12917072	ADAMTS12
cg03393607	AFF3, AC092667.2
cg01821226	PXDN
cg05955301	PRELP
cg27470554	FCGR2A
cg06238491	LAIR1
cg22695532	RP11-475O6.1
cg00742851	SUMF1, LRRN1
cg27553626	PPP1R8
cg25394505	INPP5A
cg08735211	XXbacBPG181M17.5, HLA-DMA
cg09983885	TRIM21
cg26514080	KIAA1522
cg05886789	PLXDC2
cg05826823	CIZ1, DNM1
cg20367923	XXyac-YX65C7_A.2, THBS2
cg24023498	NR4A2
cg16239257	LTBP2
cg17331738	NES

**Table A3:** Top features detected by permutation analysis from Random Survival Forest using PFS selected by 338 CpG probes.

Feature	Weight	std	Gene	Direction
cg02458945	0.00167149	0.0004095	MMP2	Positive
cg03478249	0.00245778	0.00088256	EPSTI1	Positive
cg04131610	0.00323843	0.00146985	CCR5, RP11-24F11.2	Positive
cg04217515	0.00200311	0.00073152	ITGB2	Positive
cg04244970	0.00166519	0.00067762	SLAMF7	Positive
cg05091653	0.00216262	0.00032063	SP100	Positive
cg05887821	0.00216972	0.00032553	INPP5A	Positive
cg07623567	0.00210498	0.00037064	HLA-DMB, XXbac-BPG181M17.5	Positive
cg08612539	0.00185325	0.00028535	CTA-833B7.2, NCF4	Positive
cg09076123	0.00165326	0.0007419	NCF2, SMG7	Positive
cg10307548	0.00294971	0.00098171	SOD3	Positive
cg10330169	0.00418932	0.00097532	DIS3L2	Positive
cg11197101	0.00227763	0.00036055	KIAA1522	Positive
cg13865810	0.00176505	0.00045972	COL15A1, RP11-92C4.6	Positive
cg16436782	0.00390891	0.00123599	RP11-212E4.1, COL4A1	Positive
cg17331738	0.00264999	0.00031466	NES	Positive
cg19722814	0.00184012	0.00034522	SERPINE1	Positive
cg21475610	0.00337569	0.00100237	CCNG2	Positive
cg24192663	0.00203206	0.00052522	HSPA6, RP11-25K21.6, FCGR2A	Positive
cg24815934	0.00283711	0.00098436	ITGB2	Positive
cg00450164	−2.33 $\times \;10^{-5}$	0.00011302	TRAF3IP3	Negative
cg00532319	−5.25 $\times \;10^{-5}$	9.97 $\times \;10^{-5}$	RPN1	Negative
cg00539174	−1.10 $\times \;10^{-5}$	0.0001254	CTSZ	Negative
cg01623438	−0.0001147	0.00016634	CTSZ	Negative
cg23008352	−6.65 $\times \;10^{-5}$	0.00011621	COL4A1	Negative

**Table A4:** Top features detected by permutation analysis from Random Survival Forest using OS interval selected by 338 CpG probes.

Feature	Weight	std	Gene	Direction
cg01436254	−0.0003291	0.00023658	CD86	Negative
cg03006477	−0.000143	0.00015906	CD109	Negative
cg03970350	−0.0002618	0.0001385	PES1, TCN2	Negative
cg04098585	−0.0002341	8.09 $\times 10^{-5}$	CD28	Negative
cg04131610	−0.0001756	0.00013193	CCR5, RP11-24F11.2	Negative
cg04217515	−0.0003524	0.00027475	ITGB2	Negative
cg05200628	−0.0003555	0.00013671	CD48	Negative
cg06728055	−0.0002169	0.00021168	WWTR1	Negative
cg07625783	−0.0002587	8.14 $\times 10^{-5}$	SLAMF8	Negative
cg08321366	−0.0002504	0.0001037	MMP14	Negative
cg08471739	−0.0002763	0.00018392	PLXND1	Negative
cg11800635	−0.0001998	0.00016978	DOK1, LOXL3	Negative
cg13939271	−0.0002059	0.00018542	DNM1	Negative
cg14903689	−0.0001355	0.00015751	COL18A1	Negative
cg16121744	−0.0001452	0.00012391	COL18A1	Negative
cg17859552	−0.0003278	0.00012804	INPP5A	Negative
cg19755435	−0.0001786	0.00014323	GPR65	Negative
cg22384395	−0.0002037	0.00018963	RP11-66B24.9, ALDH1A3	Negative
cg24421410	−0.0001716	6.39 $\times 10^{-5}$	XXbac-BPG181M17.5, HLA-DMA	Negative
cg26066361	−0.0001566	0.00031627	CLEC7A	Negative
cg00295382	0.00069785	0.00031361	MYCL	Positive
cg00777079	0.00194878	0.00093506	SERPINF1	Positive
cg01930947	0.00214195	0.00092059	C1orf111, RP11-565P22.6, C1orf226	Positive
cg02957057	0.00074669	0.00018464	NID1	Positive
cg03196766	0.00218507	0.00059328	THBS1	Positive
cg04297819	0.00081885	0.00047748	HSPG2	Positive
cg04499514	0.00142254	0.00078907	C3AR1	Positive
cg05091653	0.00080697	0.00048847	SP100	Positive
cg06222012	0.00135962	0.00059195	AC078941.1, AC023115.2	Positive
cg11702456	0.0028937	0.00103411	SP100	Positive
cg11827097	0.00201346	0.00095397	SP100	Positive
cg13765206	0.00158138	0.00079435	EMILIN2	Positive
cg14082886	0.00079245	0.00050107	CD44	Positive
cg14291900	0.00181238	0.00080766	SLC7A7	Positive
cg16713274	0.00066793	0.00025659	COL18A1, LL21NC02-21A1.1	Positive
cg18411043	0.0019083	0.00079707	LAPTM5	Positive
cg20640433	0.00088705	0.00032218	LAMA2	Positive
cg21218883	0.00094513	0.00049267	PRKCE	Positive
cg23986671	0.00069697	0.00025722	ADAMTS5	Positive
cg24769499	0.00116293	0.00021175	TMEM37	Positive

**Table A5:** Gene ontology (GO) that define biological function. The GO annotations, accompanied by evidence-based statements describe specific gene product and specific ontology term (biological function). g-profile enrichment terms obtained from positive and negative values of 18 CpGs selected from BECon on overal survival by LASSO procedure. All the data have *p* value low than 0.05. MF: molecular function, CC: cellular component, BP: biological process.

Source	Term_NAME	Term_id	Adjusted_*p*_Value	Negative_log10_of _Adjusted_*p*_Value	Direction Coefficient
GO:MF	glycosaminoglycan binding	GO:0005539	0.008788287085578	2.05609576449095	Negative
GO:CC	collagen-containing extracellular matrix	GO:0062023	0.048474322092945	1.31448825575832	Negative
KEGG	ECM-receptor interaction	KEGG:04512	0.037233188408127	1.42906977203396	Negative
CORUM	CD44-LRP1 complex	CORUM:7535	0.049698019554143	1.30366091738024	Negative
GO:MF	growth hormone secretagogue receptor activity	GO:0001616	0.049775611543161	1.3029833955258	Positive
GO:BP	regulation of neurotransmitter receptor localization to postsynaptic specialization membrane	GO:0098696	0.000167004924934	3.77727072142761	Positive
GO:BP	regulation of receptor localization to synapse	GO:1902683	0.001168645063196	2.93231737121765	Positive
GO:BP	protein localization to postsynaptic specialization membrane	GO:0099633	0.001335544908879	2.8743415038609	Positive
GO:BP	neurotransmitter receptor localization to postsynaptic specialization membrane	GO:0099645	0.001335544908879	2.8743415038609	Positive
GO:BP	regulation of protein localization to synapse	GO:1902473	0.003070843427737	2.51274232624767	Positive
GO:BP	protein localization to postsynaptic membrane	GO:1903539	0.007006418620425	2.15450391795907	Positive
GO:BP	protein localization to postsynapse	GO:0062237	0.009117775996435	2.04011108165666	Positive
GO:BP	response to dexamethasone	GO:0071548	0.009117775996435	2.04011108165666	Positive
GO:BP	regulation of postsynaptic membrane neurotransmitter receptor levels	GO:0099072	0.013616524667261	1.86593372285098	Positive
GO:BP	receptor localization to synapse	GO:0097120	0.013616524667261	1.86593372285098	Positive
GO:BP	protein localization to synapse	GO:0035418	0.033345288657273	1.47696551870962	Positive

**Table A6:** Feature selection with the coefficient value and gene name of CpG probe selected by LASSO COXNET in the context of the developed signature for liquid biopsy based on BECon.

CpG	COEFFICIENT	GENE	INTERVAL
cg01320433	−0.1592041	XXyac-YX65C7_A.2, THBS2	PFS
cg01508380	−0.33756425	MMP14	PFS
cg06222012	−0.5359208	AC078941.1, AC023115.2	PFS
cg06728055	−0.07162224	WWTR1	PFS
cg11029367	−0.30043008	HEG1	PFS
cg13371976	−1.39825624	PRELP	PFS
cg22716262	−0.04181589	MPP7	PFS
cg26066361	−0.17464321	CLEC7A	PFS
cg01320433	−0.37578276	XXyac-YX65C7_A.2, THBS2	OS
cg02744249	−0.5024627	CTSZ	OS
cg04244970	−0.11253157	SLAMF7	OS
cg04851268	0.96144542	GHSR	OS
cg06222012	−1.06780331	AC078941.1, AC023115.2	OS
cg07438421	0.31295409	SERPINF1	OS
cg08612539	−0.90719367	CTA-833B7.2, NCF4	OS
cg08655071	−0.10973404	TRAF3IP3	OS
cg10949632	0.5123932	GPC6	OS
cg13371976	−1.01620416	PRELP	OS
cg14082886	−1.13578356	CD44	OS
cg14943796	0.65287911	BAHCC1	OS
cg18595867	0.93613126	FOXD2-AS1	OS
cg20367923	−0.03248293	XXyac-YX65C7_A.2, THBS2	OS
cg22116670	−0.75216037	CTB-113P19.1, SPARC	OS
cg22695532	−0.67502749	RP11-475O6.1	OS
cg26066361	−1.14858784	CLEC7A	OS
cg26350754	0.88852671	HLA-DPA1, HLA-DPB1	OS

**Table A7:** Correlation of CpG probes with genes that have high correlation values from 338 CpGs used to create the model.

CpG	geneCpG	Gene	rho	*p* Value	Correlation Strength
cg13353679	AFF3, AC092667.2	HAVCR2	0.67520241	2.65 $\times \;10^{-7}$	high
cg13353679	AFF3, AC092667.2	CCR4	0.62698564	3.13 $\times \;10^{-6}$	high
cg13353679	AFF3, AC092667.2	TGFB1	0.72533605	1.19 $\times \;10^{-8}$	high
cg13353679	AFF3, AC092667.2	IL10	0.75708106	1.14 $\times \;10^{-9}$	high
cg22568423	MYO1F	HAVCR2	0.6391164	1.75 $\times \;10^{-6}$	high
cg22568423	MYO1F	TGFB1	0.61936567	4.45 $\times \;10^{-6}$	high
cg22568423	MYO1F	IL10	0.65815888	6.66 $\times \;10^{-7}$	high
cg17599241	VCAN-AS1, VCAN	HAVCR2	0.6225838	3.84 $\times \;10^{-6}$	high
cg17599241	VCAN-AS1, VCAN	TGFB1	0.66056385	5.87 $\times \;10^{-7}$	high
cg17599241	VCAN-AS1, VCAN	IL10	0.71015235	3.25 $\times \;10^{-8}$	high
cg08064683	FAT1	CCR4	0.60946563	6.94 $\times \;10^{-6}$	high
cg08064683	FAT1	TGFB1	0.7482646	2.26 $\times \;10^{-9}$	high
cg08064683	FAT1	IL10	0.66059345	5.86 $\times \;10^{-7}$	high
cg00799121	ADAMTS2	HAVCR2	0.6833261	1.67 $\times \;10^{-7}$	high
cg00799121	ADAMTS2	TGFB1	0.69708683	7.37 $\times \;10^{-8}$	high
cg00799121	ADAMTS2	IL10	0.71008968	3.26 $\times \;10^{-8}$	high
cg04153551	FBLN5	HAVCR2	0.6642975	4.81 $\times \;10^{-7}$	high
cg04153551	FBLN5	CCR4	0.63922606	1.74 $\times \;10^{-6}$	high
cg04153551	FBLN5	TGFB1	0.64462765	1.33 $\times \;10^{-6}$	high
cg04153551	FBLN5	IL10	0.75654189	1.19 $\times \;10^{-9}$	high
cg22704788	PRELP	HAVCR2	0.66998995	3.53 $\times \;10^{-7}$	high
cg22704788	PRELP	TGFB1	0.64874971	1.08 $\times \;10^{-6}$	high
cg22704788	PRELP	IL10	0.71501411	2.37 $\times \;10^{-8}$	high
cg12613839	ADAMTS2	HAVCR2	0.70546862	4.38 $\times \;10^{-8}$	high
cg12613839	ADAMTS2	TGFB1	0.70966683	3.35 $\times \;10^{-8}$	high
cg12613839	ADAMTS2	PDCD1LG2	0.60313469	9.15 $\times \;10^{-6}$	high
cg12613839	ADAMTS2	IL10	0.7323874	7.25 $\times \;10^{-9}$	high
cg02189760	CTC-301O7.4, CD37	HAVCR2	0.64276143	1.46 $\times \;10^{-6}$	high
cg02189760	CTC-301O7.4, CD37	TGFB1	0.65060293	9.85 $\times \;10^{-7}$	high
cg02189760	CTC-301O7.4, CD37	IL10	0.68557785	1.46 $\times \;10^{-7}$	high
cg07947930	PRELP	HAVCR2	0.66424332	4.82 $\times \;10^{-7}$	high
cg07947930	PRELP	TGFB1	0.68739449	1.32 $\times \;10^{-7}$	high
cg07947930	PRELP	PDCD1LG2	0.62213951	3.92 $\times \;10^{-6}$	high
cg07947930	PRELP	IL10	0.6692665	3.68 $\times \;10^{-7}$	high
cg27329371	ALDH3A1	HAVCR2	0.63023007	2.68 $\times \;10^{-6}$	high
cg27329371	ALDH3A1	TGFB1	0.666298	4.32 $\times \;10^{-7}$	high
cg27329371	ALDH3A1	PDCD1LG2	0.6318309	2.49 $\times \;10^{-6}$	high
cg27329371	ALDH3A1	IL10	0.69220582	9.90 $\times \;10^{-8}$	high
cg25206536	MIR572	HAVCR2	0.65967075	6.15 $\times \;10^{-7}$	high
cg25206536	MIR572	TGFB1	0.65401204	8.27 $\times \;10^{-7}$	high
cg25206536	MIR572	IL10	0.71059003	3.16 $\times \;10^{-8}$	high
cg20502977	COL6A3	IL10	0.66111585	5.70 $\times \;10^{-7}$	high
cg18397405	GPC6	CTLA4	0.62938967	2.79 $\times \;10^{-6}$	high
cg18397405	GPC6	HAVCR2	0.64510535	1.30 $\times \;10^{-6}$	high
cg18397405	GPC6	CCR4	0.65623064	7.37 $\times \;10^{-7}$	high
cg18397405	GPC6	TGFB1	0.75578234	1.26 $\times \;10^{-9}$	high
cg18397405	GPC6	IL10	0.76613612	5.48 $\times \;10^{-10}$	high
cg16121744	COL18A1	HAVCR2	0.76562289	5.72 $\times \;10^{-10}$	high
cg16121744	COL18A1	TGFB1	0.72925722	9.04 $\times \;10^{-9}$	high
cg16121744	COL18A1	IL10	0.77214535	3.31 $\times \;10^{-10}$	high
cg15254671	MYO1F	HAVCR2	0.73827055	4.76 $\times \;10^{-9}$	high
cg15254671	MYO1F	IL10	0.70599224	4.24 $\times \;10^{-8}$	high
cg22595235	SUMF1, LRRN1	CTLA4	0.61078204	6.55 $\times \;10^{-6}$	high
cg09777237	ELN	HAVCR2	0.67250205	3.08 $\times \;10^{-7}$	high
cg09777237	ELN	TGFB1	0.61588628	5.21 $\times \;10^{-6}$	high
cg09777237	ELN	IL10	0.68055003	1.96 $\times \;10^{-7}$	high
cg21475610	CCNG2	TGFB1	0.68792815	1.28 $\times \;10^{-7}$	high
cg21475610	CCNG2	IL10	0.6470373	1.18 $\times \;10^{-6}$	high
cg11076970	HLA-DOA	CCL22	0.68567625	1.46 $\times \;10^{-7}$	high
cg17611512	COL18A1, COL18A1-AS1	HAVCR2	0.67446387	2.76 $\times \;10^{-7}$	high
cg17611512	COL18A1, COL18A1-AS1	TGFB1	0.75562488	1.28 $\times \;10^{-9}$	high
cg17611512	COL18A1, COL18A1-AS1	IL10	0.76622066	5.44 $\times \;10^{-10}$	high
cg22987448	MYO1F	HAVCR2	0.70835256	3.65 $\times \;10^{-8}$	high
cg22987448	MYO1F	TGFB1	0.63248797	2.41 $\times \;10^{-6}$	high
cg22987448	MYO1F	IL10	0.70835142	3.65 $\times \;10^{-8}$	high
cg21398469	CCNG2	TGFB1	0.61126793	6.41 $\times \;10^{-6}$	high
cg05955301	PRELP	HAVCR2	0.64915158	1.06 $\times \;10^{-6}$	high
cg05955301	PRELP	TGFB1	0.658497	6.55 $\times \;10^{-7}$	high
cg05955301	PRELP	IL10	0.69042987	1.10 $\times \;10^{-7}$	high
cg00742851	SUMF1, LRRN1	HAVCR2	0.64108721	1.59 $\times \;10^{-6}$	high
cg00742851	SUMF1, LRRN1	CCR4	0.65102622	9.64 $\times \;10^{-7}$	high
cg00742851	SUMF1, LRRN1	TGFB1	0.73293612	6.98 $\times \;10^{-9}$	high
cg00742851	SUMF1, LRRN1	IL10	0.75167928	1.74 $\times \;10^{-9}$	high

**Table A8:** Correlation among 338 CpG probes with gene expression of paired sample that present a high correlation value and significant *p* value.

CpG	Gene	rho	*p* Value	CpGgene	Magnitude
cg02957057	DEFB126	0.99984824	$4.85\;\times \;10^{-79}$	NID1	high
cg20640433	LRRIQ4	−0.8429582	$2.00\;\times \;10^{-13}$	LAMA2	high
cg02957057	ZDHHC8P1	−0.8399142	$2.96\;\times \;10^{-13}$	NID1	high
cg23986671	KRTAP6-3	0.83840023	$3.58\;\times \;10^{-13}$	ADAMTS5	high
cg20640433	TXK	−0.8184023	$3.75\;\times \;10^{-12}$	LAMA2	high
cg20640433	NLRP14	−0.8100527	$9.19\;\times \;10^{-12}$	LAMA2	high
cg20640433	DEFB126	0.80488085	$1.57\;\times \;10^{-11}$	LAMA2	high
cg16713274	OR56A5	0.79052422	$6.38\;\times \;10^{-11}$	COL18A1	high
cg02957057	RFESD	−0.7883283	$7.83\;\times \;10^{-11}$	NID1	high
cg02957057	MMACHC	−0.7871875	$8.70\;\times \;10^{-11}$	NID1	high
cg20640433	GRP	−0.7852917	$1.04\;\times \;10^{-10}$	LAMA2	high
cg02957057	ISPD	−0.7804697	$1.60\times 10^{-10}$	NID1	high
cg02957057	OSBPL9	−0.7789042	$1.84\;\times \;10^{-10}$	NID1	high
cg20640433	FBXO17	−0.7746792	$2.66\;\times \;10^{-10}$	LAMA2	high
cg16121744	IL10	0.77214535	$3.31\;\times \;10^{-10}$	COL18A1	high
cg18397405	CCR5	0.76767048	$4.82\;\times \;10^{-10}$	GPC6	high
cg18397405	CD96	0.7668386	$5.17\;\times \;10^{-10}$	GPC6	high
cg17611512	IL10	0.76622066	$5.44\;\times \;10^{-10}$	COL18A1	high
cg18397405	IL10	0.76613612	$5.48\;\times \;10^{-10}$	GPC6	high
cg16121744	HAVCR2	0.76562289	$5.72\;\times \;10^{-10}$	COL18A1	high
cg02957057	PCGEM1	0.76170335	$7.88\;\times \;10^{-10}$	NID1	high
cg02957057	ANKRD7	−0.7606609	$8.57\;\times \;10^{-10}$	NID1	high
cg13353679	IL10	0.75708106	$1.14\;\times \;10^{-9}$	AFF3, AC092667.2	high
cg04153551	IL10	0.75654189	$1.19\;\times \;10^{-9}$	FBLN5	high
cg18397405	TGFB1	0.75578234	$1.26\;\times \;10^{-9}$	GPC6	high
cg17611512	TGFB1	0.75562488	$1.28\;\times \;10^{-9}$	COL18A1, COL18A1-AS1	high
cg18397405	ITGB2	0.75480536	$1.37\;\times \;10^{-9}$	GPC6	high
cg20640433	FAHD2B	−0.7544591	$1.40\;\times \;10^{-9}$	LAMA2	high
cg00742851	IL10	0.75167928	$1.74\;\times \;10^{-9}$	SUMF1, LRRN1	high
cg23986671	TAF1L	−0.7511669	$1.81\;\times \;10^{-9}$	ADAMTS5	high
cg08064683	TGFB1	0.7482646	$2.26\;\times \;10^{-9}$	FAT1	high
cg20640433	IL22RA1	−0.7447273	$2.96\;\times \;10^{-9}$	LAMA2	high
cg18411043	GIMAP5	0.74431857	$3.05\;\times \;10^{-9}$	LAPTM5	high
cg02957057	FRMPD2	−0.7389076	$4.54\;\times \;10^{-9}$	NID1	high
cg02957057	FAHD2B	−0.7387551	$4.59\;\times \;10^{-9}$	NID1	high
cg15254671	HAVCR2	0.73827055	$4.76\;\times \;10^{-9}$	MYO1F	high
cg18397405	CD163	0.73748155	$5.04\;\times \;10^{-9}$	GPC6	high
cg16713274	C6orf132	−0.7366047	$5.37\;\times \;10^{-9}$	COL18A1	high
cg20640433	ZDHHC8P1	−0.7353041	$5.89\;\times \;10^{-9}$	LAMA2	high
cg02957057	MAP1LC3A	−0.7341328	$6.41\;\times \;10^{-9}$	NID1	high
cg00742851	TGFB1	0.73293612	$6.98\;\times \;10^{-9}$	SUMF1, LRRN1	high
cg12613839	IL10	0.7323874	$7.25\;\times \;10^{-9}$	ADAMTS2	high
cg18411043	WDR76	−0.7298891	$8.65\;\times \;10^{-9}$	LAPTM5	high
cg16121744	TGFB1	0.72925722	$9.04\;\times \;10^{-9}$	COL18A1	high
cg18411043	SALL3	−0.7280974	$9.80\;\times \;10^{-9}$	LAPTM5	high
cg20640433	GUCY2D	−0.7279513	$9.90\;\times \;10^{-9}$	LAMA2	high
cg20640433	ALDH7A1	−0.7273038	$1.04\;\times \;10^{-8}$	LAMA2	high
cg13353679	TGFB1	0.72533605	$1.19\;\times \;10^{-8}$	AFF3, AC092667.2	high
cg18397405	CD74	0.72520583	$1.20\;\times \;10^{-8}$	GPC6	high
cg14291900	SFMBT2	0.72226357	$1.46\;\times \;10^{-8}$	SLC7A7	high
cg22704788	IL10	0.71501411	$2.37\;\times \;10^{-8}$	PRELP	high
cg02957057	DPEP3	−0.7149296	$2.38\;\times \;10^{-8}$	NID1	high
cg20640433	C17orf82	−0.7134266	$2.63\;\times \;10^{-8}$	LAMA2	high
cg18411043	KCNK6	0.71200028	$2.89\;\times \;10^{-8}$	LAPTM5	high
cg02957057	N6AMT2	−0.7119956	$2.89\;\times \;10^{-8}$	NID1	high
cg02957057	SLC25A20	−0.7113793	$3.00\;\times \;10^{-8}$	NID1	high
cg18397405	CD14	0.71124306	$3.03\;\times \;10^{-8}$	GPC6	high
cg25206536	IL10	0.71059003	$3.16\;\times \;10^{-8}$	MIR572	high
cg02957057	ITPRIPL1	−0.7102111	$3.24\;\times \;10^{-8}$	NID1	high
cg17599241	IL10	0.71015235	$3.25\;\times \;10^{-8}$	VCAN-AS1, VCAN	high
cg00799121	IL10	0.71008968	$3.26\;\times \;10^{-8}$	ADAMTS2	high
cg20640433	SVOPL	−0.7100842	$3.27\;\times \;10^{-8}$	LAMA2	high
cg12613839	TGFB1	0.70966683	$0.35\;\times \;10^{-8}$	ADAMTS2	high
cg22987448	HAVCR2	0.70835256	$3.65\;\times \;10^{-8}$	MYO1F	high
cg22987448	IL10	0.70835142	$3.65\;\times \;10^{-8}$	MYO1F	high
cg18397405	CD68	0.70783649	$3.77\;\times \;10^{-8}$	GPC6	high
cg18411043	TGFBR2	0.70621641	$4.18\;\times \;10^{-8}$	LAPTM5	high
cg15254671	IL10	0.70599224	$4.24\;\times \;10^{-8}$	MYO1F	high
cg12613839	HAVCR2	0.70546862	$4.38\;\times \;10^{-8}$	ADAMTS2	high
cg04499514	PDIA6	−0.7048045	$4.57\;\times \;10^{-8}$	C3AR1	high
cg20640433	C9orf64	−0.7042363	$4.74\;\times \;10^{-8}$	LAMA2	high
cg02957057	SLC35F3	−0.7035707	$4.94\;\times \;10^{-8}$	NID1	high
cg02957057	POTEA	0.70296616	$5.13\;\times \;10^{-8}$	NID1	high
cg18411043	IGFBP6	0.70161792	$5.58\;\times \;10^{-8}$	LAPTM5	high
cg23986671	TWIST2	−0.7011775	$5.73\;\times \;10^{-8}$	ADAMTS5	high
cg02957057	OR10G7	0.70091288	$5.83\;\times \;10^{-8}$	NID1	high
cg14291900	FGD3	0.70047149	$5.99\;\times \;10^{-8}$	SLC7A7	high
cg18397405	GPR65	0.70013203	$6.11\;\times \;10^{-8}$	GPC6	high
cg23986671	MYOZ2	−0.6993028	$6.43\;\times \;10^{-8}$	ADAMTS5	high
cg23986671	PDE6C	−0.6980551	$6.95\;\times \;10^{-8}$	ADAMTS5	high
cg00799121	TGFB1	0.69708683	$7.37\;\times \;10^{-8}$	ADAMTS2	high
cg20640433	AREG	−0.6964744	$7.65\;\times \;10^{-8}$	LAMA2	high
cg20640433	NMNAT3	−0.6951494	$8.29\;\times \;10^{-8}$	LAMA2	high
cg20640433	XKR8	−0.6950749	$8.33\;\times \;10^{-8}$	LAMA2	high
cg20640433	SLC25A44	0.69436599	$8.69\;\times \;10^{-8}$	LAMA2	high
cg02957057	ANKK1	−0.6934215	$9.20\;\times \;10^{-8}$	NID1	high
cg18397405	GRN	0.69317267	$9.34\;\times \;10^{-8}$	GPC6	high
cg18411043	TNFRSF10D	0.6931024	$9.38\;\times \;10^{-8}$	LAPTM5	high
cg18411043	KIF22	−0.6926606	$9.63\;\times \;10^{-8}$	LAPTM5	high
cg20640433	HDHD3	−0.6922066	$9.90\;\times \;10^{-8}$	LAMA2	high
cg27329371	IL10	0.69220582	$9.90\;\times \;10^{-8}$	ALDH3A1	high
cg18411043	C19orf57	−0.6912927	$1.05\;\times \;10^{-7}$	LAPTM5	high
cg18411043	SERPINB9	0.69077738	$1.08\;\times \;10^{-7}$	LAPTM5	high
cg05955301	IL10	0.69042987	$1.10\;\times \;10^{-7}$	PRELP	high
cg18411043	CACNA2D4	0.6878496	$1.28\;\times \;10^{-7}$	LAPTM5	high
cg21475610	TGFB1	0.68792815	$1.28\;\times \;10^{-7}$	CCNG2	high
cg20640433	SSH3	−0.6876975	$1.29\;\times \;10^{-7}$	LAMA2	high
cg07947930	TGFB1	0.68739449	$1.32\;\times \;10^{-7}$	PRELP	high
cg18411043	CLDN23	0.68678752	$1.36\;\times \;10^{-7}$	LAPTM5	high
cg02957057	ZNF683	−0.6867046	$1.37\;\times \;10^{-7}$	NID1	high
cg02189760	IL10	0.68557785	$1.46\;\times \;10^{-7}$	CTC-301O7.4, CD37	high
cg11076970	CCL22	0.68567625	$1.46\;\times \;10^{-7}$	HLA-DOA	high
cg13765206	AMN	−0.6852093	$1.50\;\times \;10^{-7}$	EMILIN2	high
cg02957057	ISG20L2	0.68458461	$1.55\;\times \;10^{-7}$	NID1	high
cg20640433	MAP1LC3A	−0.683593	$1.64\;\times \;10^{-7}$	LAMA2	high
cg18397405	CCL5	0.68360635	$1.64\;\times \;10^{-7}$	GPC6	high
cg00799121	HAVCR2	0.6833261	$1.67\;\times \;10^{-7}$	ADAMTS2	high
cg18411043	MKS1	−0.6816571	$1.84\;\times \;10^{-7}$	LAPTM5	high
cg18411043	IL4R	0.68111791	$1.89\;\times \;10^{-7}$	LAPTM5	high
cg09777237	IL10	0.68055003	$1.96\;\times \;10^{-7}$	ELN	high
cg18411043	GIMAP6	0.68005964	$2.01\;\times \;10^{-7}$	LAPTM5	high
cg02957057	STK33	−0.6799005	$2.03\;\times \;10^{-7}$	NID1	high
cg02957057	PYDC2	0.67988313	$2.03\;\times \;10^{-7}$	NID1	high
cg20640433	MYD88	−0.6798431	$2.04\;\times \;10^{-7}$	LAMA2	high
cg14291900	PIK3IP1	0.6795888	2.07$\;\times \;10^{-7}$	SLC7A7	high
cg18411043	NUSAP1	−0.6794355	2.08$\;\times \;10^{-7}$	LAPTM5	high
cg23986671	RFPL3S	−0.6794146	2.09$\;\times \;10^{-7}$	ADAMTS5	high
cg20640433	HEBP1	−0.6789805	2.14$\;\times \;10^{-7}$	LAMA2	high
cg04499514	RUNX1	−0.6782038	2.23$\;\times \;10^{-7}$	C3AR1	high
cg18397405	GZMA	0.67785253	2.28$\;\times \;10^{-7}$	GPC6	high
cg02957057	FAM19A1	−0.6772002	2.37$\;\times \;10^{-7}$	NID1	high
cg02957057	SPRR1A	0.67668058	2.44$\;\times \;10^{-7}$	NID1	high
cg20640433	MSN	−0.6761721	2.51$\;\times \;10^{-7}$	LAMA2	high
cg11827097	PTK6	0.67530072	2.63$\;\times \;10^{-7}$	SP100	high
cg13353679	HAVCR2	0.67520241	2.65$\;\times \;10^{-7}$	AFF3, AC092667.2	high
cg20640433	SH3RF2	−0.6749581	2.68$\;\times \;10^{-7}$	LAMA2	high
cg17611512	HAVCR2	0.67446387	2.76$\;\times \;10^{-7}$	COL18A1, COL18A1-AS1	high
cg20640433	PACSIN3	−0.6738627	2.85$\;\times \;10^{-7}$	LAMA2	high
cg02957057	CMBL	−0.673238	2.95$\;\times \;10^{-7}$	NID1	high
cg18411043	MCTP2	0.67300302	2.99$\;\times \;10^{-7}$	LAPTM5	high
cg07436701	CD74	−0.6729908	2.99$\;\times \;10^{-7}$	MMRN2, SNCG	high
cg14082886	PPP1R15A	−0.6727144	3.04$\;\times \;10^{-7}$	CD44	high
cg18411043	NCAPD3	−0.6726225	3.06$\;\times \;10^{-7}$	LAPTM5	high
cg09777237	HAVCR2	0.67250205	3.08$\;\times \;10^{-7}$	ELN	high
cg02957057	TYSND1	−0.6716086	3.23$\;\times \;10^{-7}$	NID1	high
cg04499514	TSPO	−0.6709273	3.35$\;\times \;10^{-7}$	C3AR1	high
cg18411043	TMEM87B	0.67084118	3.37$\;\times \;10^{-7}$	LAPTM5	high
cg23986671	ZBTB32	−0.6707326	3.39$\;\times \;10^{-7}$	ADAMTS5	high
cg14291900	ZNF71	−0.669991	3.53$\;\times \;10^{-7}$	SLC7A7	high
cg22704788	HAVCR2	0.66998995	3.53$\;\times \;10^{-7}$	PRELP	high
cg18411043	B3GNT2	0.66993926	3.54$\;\times \;10^{-7}$	LAPTM5	high
cg07947930	IL10	0.6692665	3.68$\;\times \;10^{-7}$	PRELP	high
cg18411043	MAPK13	0.66886709	3.76$\;\times \;10^{-7}$	LAPTM5	high
cg20640433	SHROOM1	−0.6687824	3.77$\;\times \;10^{-7}$	LAMA2	high
cg14291900	ZNF134	−0.6665228	4.27$\;\times \;10^{-7}$	SLC7A7	high
cg27329371	TGFB1	0.666298	4.32$\;\times \;10^{-7}$	ALDH3A1	high
cg07436701	CCR5	−0.6662434	4.33$\;\times \;10^{-7}$	MMRN2, SNCG	high
cg18411043	CYP1B1	0.66507974	4.61$\;\times \;10^{-7}$	LAPTM5	high
cg18411043	EMB	0.66449718	4.76$\;\times \;10^{-7}$	LAPTM5	high
cg04153551	HAVCR2	0.6642975	4.81$\;\times \;10^{-7}$	FBLN5	high
cg07947930	HAVCR2	0.66424332	4.82$\;\times \;10^{-7}$	PRELP	high
cg04499514	MAPT	0.66400429	4.89$\;\times \;10^{-7}$	C3AR1	high
cg13765206	ITCH	0.66292628	5.18$\;\times \;10^{-7}$	EMILIN2	high
cg20640433	RFESD	−0.6627852	5.22$\;\times \;10^{-7}$	LAMA2	high
cg14082886	CLVS2	0.66221087	5.38$\;\times \;10^{-7}$	CD44	high
cg18411043	CHEK1	−0.6614723	$5.59\;\times \;10^{-7}$	LAPTM5	high
cg11702456	TAGLN2	−0.6614648	$5.60\;\times \;10^{-7}$	SP100	high
cg18397405	CD244	0.66144045	$5.60\;\times \;10^{-7}$	GPC6	high
cg20502977	IL10	0.66111585	$5.70\;\times \;10^{-7}$	COL6A3	high
cg18411043	PAPSS2	0.66103523	$5.73\;\times \;10^{-7}$	LAPTM5	high
cg00295382	MKRN3	−0.6607885	$5.80\;\times \;10^{-7}$	MYCL	high
cg08064683	IL10	0.66059345	$5.86\;\times \;10^{-7}$	FAT1	high
cg17599241	TGFB1	0.66056385	$5.87\;\times \;10^{-7}$	VCAN-AS1, VCAN	high
cg23986671	GCOM1	−0.660442	$5.91\;\times \;10^{-7}$	ADAMTS5	high
cg18411043	LYVE1	0.6597996	$6.11\;\times \;10^{-7}$	LAPTM5	high
cg25206536	HAVCR2	0.65967075	$6.15\;\times \;10^{-7}$	MIR572	high
cg14082886	RGS9	0.65942308	$6.24\;\times \;10^{-7}$	CD44	high
cg14082886	NEK6	−0.6591273	$6.33\;\times \;10^{-7}$	CD44	high
cg18411043	NUMB	0.65897549	$6.38\;\times \;10^{-7}$	LAPTM5	high
cg20640433	SLC43A3	−0.6588459	$6.43\;\times \;10^{-7}$	LAMA2	high
cg23986671	VTCN1	−0.6588003	$6.44\;\times \;10^{-7}$	ADAMTS5	high
cg20640433	RFPL2	−0.6584724	$6.55\;\times \;10^{-7}$	LAMA2	high
cg05955301	TGFB1	0.658497	$6.55\;\times \;10^{-7}$	PRELP	high
cg22568423	IL10	0.65815888	$6.66\;\times \;10^{-7}$	MYO1F	high
cg18397405	CCR4	0.65623064	$7.37\;\times \;10^{-7}$	GPC6	high
cg18397405	CCR4	0.65623064	$7.37\;\times \;10^{-7}$	GPC6	high
cg14291900	YPEL2	0.65585997	$7.51\;\times \;10^{-7}$	SLC7A7	high
cg20640433	ZDHHC1	−0.6557248	$7.57\;\times \;10^{-7}$	LAMA2	high
cg18411043	MAP3K8	0.6551703	$7.79\;\times \;10^{-7}$	LAPTM5	high
cg02957057	HSD17B7	−0.6541632	$8.21\;\times \;10^{-7}$	NID1	high
cg25206536	TGFB1	0.65401204	$8.27\;\times \;10^{-7}$	MIR572	high
cg18411043	GAB1	−0.6539384	$8.30\;\times \;10^{-7}$	LAPTM5	high
cg18411043	OIP5	−0.6532665	$8.59\;\times \;10^{-7}$	LAPTM5	high
cg04499514	LGALS1	−0.6531144	$8.66\;\times \;10^{-7}$	C3AR1	high
cg23986671	HYALP1	0.65295592	$8.73\;\times \;10^{-7}$	ADAMTS5	high
cg02957057	SCAMP3	0.65296148	$8.73\;\times \;10^{-7}$	NID1	high
cg20640433	DYNLT3	−0.6527851	$8.81\;\times \;10^{-7}$	LAMA2	high
cg04499514	CD63	−0.6526897	$8.85\;\times \;10^{-7}$	C3AR1	high
cg04499514	CD63	−0.6526897	$8.85\;\times \;10^{-7}$	C3AR1	high
cg18411043	HIST1H4A	−0.652456	$8.96\;\times \;10^{-7}$	LAPTM5	high
cg18397405	IGF1	0.65236854	$9.00\;\times \;10^{-7}$	GPC6	high
cg14291900	ZNF787	−0.6523244	$9.02\;\times \;10^{-7}$	SLC7A7	high
cg20640433	SH2D4A	−0.6513244	$9.50\;\times \;10^{-7}$	LAMA2	high
cg23986671	MMP1	−0.6510743	$9.62\;\times \;10^{-7}$	ADAMTS5	high
cg07436701	ITGB2	−0.6510564	$9.63\;\times \;10^{-7}$	MMRN2, SNCG	high
cg00742851	CCR4	0.65102622	$9.64\;\times \;10^{-7}$	SUMF1, LRRN1	high
cg04499514	RPS6KA5	0.65061879	$9.84\;\times \;10^{-7}$	C3AR1	high
cg02189760	TGFB1	0.65060293	$9.85\;\times \;10^{-7}$	CTC-301O7.4, CD37	high
cg18411043	CD59	0.65051987	$9.89\;\times \;10^{-7}$	LAPTM5	high
cg18411043	ST3GAL1	0.64986187	$1.02\;\times \;10^{-6}$	LAPTM5	high
cg18411043	ZNF620	−0.6492829	$1.05\;\times \;10^{-6}$	LAPTM5	high
cg04499514	CRELD2	−0.6492545	$1.06\;\times \;10^{-6}$	C3AR1	high
cg05955301	HAVCR2	0.64915158	$1.06\;\times \;10^{-6}$	PRELP	high
cg20640433	ACSF2	−0.6487996	$1.08\;\times \;10^{-6}$	LAMA2	high
cg02957057	PARVA	−0.6487478	$1.08\;\times \;10^{-6}$	NID1	high
cg22704788	TGFB1	0.64874971	$1.08\;\times \;10^{-6}$	PRELP	high
cg16713274	BCL2L10	−0.6485899	$1.09\;\times \;10^{-6}$	COL18A1	high
cg20640433	SLC35F3	−0.6482366	$1.11\;\times \;10^{-6}$	LAMA2	high
cg14291900	KLHL32	0.6481981	$1.11\;\times \;10^{-6}$	SLC7A7	high
cg11702456	RIPK1	−0.6478933	$1.13\;\times \;10^{-6}$	SP100	high
cg11702456	PTK6	0.64795892	$1.13\;\times \;10^{-6}$	SP100	high
cg14291900	ZNF473	−0.6477937	$1.14\;\times \;10^{-6}$	SLC7A7	high
cg13765206	CRNKL1	0.64734872	$1.16\;\times \;10^{-6}$	EMILIN2	high
cg14291900	AKAP8	−0.6473027	$1.16\;\times \;10^{-6}$	SLC7A7	high
cg18411043	PSTPIP2	0.64723289	$1.17\;\times \;10^{-6}$	LAPTM5	high
cg21475610	IL10	0.6470373	$1.18\;\times \;10^{-6}$	CCNG2	high
cg11702456	RAB34	−0.6468892	$1.19\;\times \;10^{-6}$	SP100	high
cg02957057	XKR8	−0.6468834	$1.19\;\times \;10^{-6}$	NID1	high
cg18411043	LTBP2	0.64651112	$1.21\;\times \;10^{-6}$	LAPTM5	high
cg18411043	WHSC1	−0.6464405	$1.22\;\times \;10^{-6}$	LAPTM5	high
cg04499514	SMAGP	−0.6459406	$1.25\;\times \;10^{-6}$	C3AR1	high
cg11827097	RIPK1	−0.6456769	$1.26\;\times \;10^{-6}$	SP100	high
cg18411043	B4GALT1	0.64554786	$1.27\;\times \;10^{-6}$	LAPTM5	high
cg02957057	UCHL1	−0.6451568	$1.30\;\times \;10^{-6}$	NID1	high
cg18397405	HAVCR2	0.64510535	$1.30\;\times \;10^{-6}$	GPC6	high
cg11702456	EMP3	−0.6447434	$1.32\;\times \;10^{-6}$	SP100	high
cg18411043	LILRB2	0.64470941	$1.33\;\times \;10^{-6}$	LAPTM5	high
cg04153551	TGFB1	0.64462765	$1.33\;\times \;10^{-6}$	FBLN5	high
cg18411043	PLK4	−0.6445754	$1.34\;\times \;10^{-6}$	LAPTM5	high
cg18411043	TNFRSF10A	0.64435812	$1.35\;\times \;10^{-6}$	LAPTM5	high
cg13765206	HPS1	−0.6438577	$1.38\;\times \;10^{-6}$	EMILIN2	high
cg02957057	PPP1R3C	−0.6439269	$1.38\;\times \;10^{-6}$	NID1	high
cg13765206	KLHDC7B	−0.643828	$1.39\;\times \;10^{-6}$	EMILIN2	high
cg18411043	GPSM2	−0.6430751	$1.44\;\times \;10^{-6}$	LAPTM5	high
cg18411043	POLA2	−0.6428379	$1.46\;\times \;10^{-6}$	LAPTM5	high
cg02189760	HAVCR2	0.64276143	$1.46\;\times \;10^{-6}$	CTC-301O7.4, CD37	high
cg18411043	MCM2	−0.6424966	$1.48\;\times \;10^{-6}$	LAPTM5	high
cg04499514	HSP90B1	−0.6419106	$1.52\;\times \;10^{-6}$	C3AR1	high
cg14291900	HPN	0.64190596	$1.53\;\times \;10^{-6}$	SLC7A7	high
cg04499514	EMILIN2	−0.6416321	$1.55\;\times \;10^{-6}$	C3AR1	high
cg04499514	EMILIN2	−0.6416321	$1.55\;\times \;10^{-6}$	C3AR1	high
cg14082886	HSPA5	−0.6412986	$1.57\;\times \;10^{-6}$	CD44	high
cg18411043	ASGR2	0.6412585	$1.57\;\times \;10^{-6}$	LAPTM5	high
cg18411043	PRKCD	0.64119535	$1.58\;\times \;10^{-6}$	LAPTM5	high
cg00742851	HAVCR2	0.64108721	$1.59\;\times \;10^{-6}$	SUMF1, LRRN1	high
cg18411043	FAM181B	−0.6409853	$1.60\;\times \;10^{-6}$	LAPTM5	high
cg00295382	ZNF292	−0.6404944	$1.63\;\times \;10^{-6}$	MYCL	high
cg11702456	TSEN34	−0.6397343	$1.70\;\times \;10^{-6}$	SP100	high
cg04153551	CCR4	0.63922606	$1.74\;\times \;10^{-6}$	FBLN5	high
cg22568423	HAVCR2	0.6391164	$1.75\;\times \;10^{-6}$	MYO1F	high
cg04499514	SPRR2A	0.63900466	$1.76\;\times \;10^{-6}$	C3AR1	high
cg20640433	CRHR2	−0.6389645	$1.76\;\times \;10^{-6}$	LAMA2	high
cg14291900	ERMN	0.63877586	$1.78\;\times \;10^{-6}$	SLC7A7	high
cg16713274	VWDE	−0.6386629	$1.79\;\times \;10^{-6}$	COL18A1	high
cg20640433	SCAMP3	0.63863905	$1.79\;\times \;10^{-6}$	LAMA2	high
cg02957057	SMG5	0.63832232	$1.82\;\times \;10^{-6}$	NID1	high
cg18411043	CDCA5	−0.637901	$1.86\;\times \;10^{-6}$	LAPTM5	high
cg18411043	SMC2	−0.6376489	$1.88\;\times \;10^{-6}$	LAPTM5	high
cg23986671	GPS1	0.63753383	$1.89\;\times \;10^{-6}$	ADAMTS5	high
cg20640433	OR10G7	0.63739025	$1.90\;\times \;10^{-6}$	LAMA2	high
cg20640433	VNN3	−0.6368153	$1.96\;\times \;10^{-6}$	LAMA2	high
cg18411043	RNF144B	0.63671956	$1.97\;\times \;10^{-6}$	LAPTM5	high
cg02957057	NMNAT3	−0.6360154	$2.03\;\times \;10^{-6}$	NID1	high
cg18411043	FANCC	−0.6359325	$2.04\;\times \;10^{-6}$	LAPTM5	high
cg14291900	SLC46A3	0.63593824	$2.04\;\times \;10^{-6}$	SLC7A7	high
cg04499514	TSEN34	−0.6356583	$2.07\;\times \;10^{-6}$	C3AR1	high
cg14082886	PCYT1A	−0.6351289	2.12$\;\times \;10^{-6}$	CD44	high
cg18411043	ARPC1B	0.63501952	2.13$\;\times \;10^{-6}$	LAPTM5	high
cg18411043	GPR132	0.63497935	2.14$\;\times \;10^{-6}$	LAPTM5	high
cg02957057	ELOVL3	−0.6344793	2.19$\;\times \;10^{-6}$	NID1	high
cg13765206	C2CD4D	−0.6344131	2.20$\;\times \;10^{-6}$	EMILIN2	high
cg14291900	SEMA4A	0.63440849	2.20$\;\times \;10^{-6}$	SLC7A7	high
cg18411043	KIF15	−0.6340036	2.24$\;\times \;10^{-6}$	LAPTM5	high
cg18411043	NCF4	0.63389721	2.25$\;\times \;10^{-6}$	LAPTM5	high
cg23986671	DCST1	−0.6335087	2.30$\;\times \;10^{-6}$	ADAMTS5	high
cg00777079	N4BP2	−0.6335114	2.30$\;\times \;10^{-6}$	SERPINF1	high
cg23986671	CLEC4F	−0.6330094	2.35$\;\times \;10^{-6}$	ADAMTS5	high
cg04499514	DUSP4	−0.632834	2.37$\;\times \;10^{-6}$	C3AR1	high
cg14291900	TCF3	−0.6328327	2.37$\;\times \;10^{-6}$	SLC7A7	high
cg14291900	ZNF416	−0.6328258	2.37$\;\times \;10^{-6}$	SLC7A7	high
cg18411043	CD1D	0.63278019	2.38$\;\times \;10^{-6}$	LAPTM5	high
cg22987448	TGFB1	0.63248797	2.41$\;\times \;10^{-6}$	MYO1F	high
cg20640433	GLIS3	−0.6319628	2.47$\;\times \;10^{-6}$	LAMA2	high
cg04499514	SEC24D	−0.631827	2.49$\;\times \;10^{-6}$	C3AR1	high
cg02957057	NLRX1	−0.6317766	2.49$\;\times \;10^{-6}$	NID1	high
cg27329371	PDCD1LG2	0.6318309	2.49$\;\times \;10^{-6}$	ALDH3A1	high
cg18411043	PSMC3IP	−0.631709	2.50$\;\times \;10^{-6}$	LAPTM5	high
cg23986671	GOLGA4	−0.6315958	2.52$\;\times \;10^{-6}$	ADAMTS5	high
cg14291900	U2AF2	−0.6314722	2.53$\;\times \;10^{-6}$	SLC7A7	high
cg18411043	CEP72	−0.6310813	2.58$\;\times \;10^{-6}$	LAPTM5	high
cg18411043	NCAPH	−0.6308592	2.61$\;\times \;10^{-6}$	LAPTM5	high
cg18411043	TRIM38	0.63055942	2.64$\;\times \;10^{-6}$	LAPTM5	high
cg27329371	HAVCR2	0.63023007	2.68$\;\times \;10^{-6}$	ALDH3A1	high
cg04499514	S100A11	−0.6301523	2.69$\;\times \;10^{-6}$	C3AR1	high
cg18411043	GIMAP8	0.63012277	2.70$\;\times \;10^{-6}$	LAPTM5	high
cg18411043	LMNB1	−0.6298277	2.74$\;\times \;10^{-6}$	LAPTM5	high
cg04499514	SEMA3D	0.62956973	2.77$\;\times \;10^{-6}$	C3AR1	high
cg18397405	CTLA4	0.62938967	2.79$\;\times \;10^{-6}$	GPC6	high
cg14291900	DOCK5	0.62929209	2.81$\;\times \;10^{-6}$	SLC7A7	high
cg14291900	ACSM5	0.62920997	2.82$\;\times \;10^{-6}$	SLC7A7	high
cg04499514	TWF2	−0.6290565	2.84$\;\times \;10^{-6}$	C3AR1	high
cg18411043	MRC1	0.62895863	2.85$\;\times \;10^{-6}$	LAPTM5	high
cg18411043	TNFRSF1B	0.62892778	2.86$\;\times \;10^{-6}$	LAPTM5	high
cg18411043	MEN1	−0.6288512	2.87$\;\times \;10^{-6}$	LAPTM5	high
cg18411043	RAB11FIP1	0.62865829	2.89$\;\times \;10^{-6}$	LAPTM5	high
cg18411043	F13A1	0.62865476	2.89$\;\times \;10^{-6}$	LAPTM5	high
cg18411043	TESC	0.62858843	2.90$\;\times \;10^{-6}$	LAPTM5	high
cg14291900	LGALS9C	0.62857927	2.90$\;\times \;10^{-6}$	SLC7A7	high
cg18411043	GIPC2	0.62849757	2.91$\;\times \;10^{-6}$	LAPTM5	high
cg02957057	LRRIQ4	−0.6285485	2.91$\;\times \;10^{-6}$	NID1	high
cg14291900	NKAIN2	0.6284529	2.92$\;\times \;10^{-6}$	SLC7A7	high
cg01930947	TACR1	0.62834753	2.93$\;\times \;10^{-6}$	C1orf111	high
cg14082886	COL4A3	0.62833753	2.94$\;\times \;10^{-6}$	CD44	high
cg04499514	RPS6KA3	−0.6282354	2.95$\;\times \;10^{-6}$	C3AR1	high
cg14291900	LHPP	0.62822146	2.95$\;\times \;10^{-6}$	SLC7A7	high
cg11702456	GALNS	−0.6280356	2.98$\;\times \;10^{-6}$	SP100	high
cg18411043	AMICA1	0.6278709	3.00$\;\times \;10^{-6}$	LAPTM5	high
cg20640433	ISG20L2	0.62790094	3.00$\;\times \;10^{-6}$	LAMA2	high
cg13765206	PLEKHG6	−0.6278379	3.01$\;\times \;10^{-6}$	EMILIN2	high
cg04499514	TTC38	−0.6274235	3.06$\;\times \;10^{-6}$	C3AR1	high
cg20640433	RAB36	−0.627427	3.06$\;\times \;10^{-6}$	LAMA2	high
cg20640433	CST3	−0.6274226	3.06$\;\times \;10^{-6}$	LAMA2	high
cg18411043	MLKL	0.62716867	3.10$\;\times \;10^{-6}$	LAPTM5	high
cg02957057	C9orf64	−0.627194	3.10$\;\times \;10^{-6}$	NID1	high
cg11702456	S100A13	−0.626989	3.13$\;\times \;10^{-6}$	SP100	high
cg01930947	TMEFF2	0.62681323	3.15$\;\times \;10^{-6}$	C1orf111	high
cg18411043	MAN1A1	0.62676193	3.16$\;\times \;10^{-6}$	LAPTM5	high
cg02957057	FBXO17	−0.6267928	3.16$\;\times \;10^{-6}$	NID1	high
cg02957057	SH3BP2	−0.6266051	3.18$\;\times \;10^{-6}$	NID1	high
cg05091653	SERPINF2	−0.6263606	3.22$\;\times \;10^{-6}$	SP100	high
cg18411043	TRPM2	0.62624593	3.24$\;\times \;10^{-6}$	LAPTM5	high
cg18411043	CD33	0.62613919	3.25$\;\times \;10^{-6}$	LAPTM5	high
cg18411043	CD46	0.62591061	3.29$\;\times \;10^{-6}$	LAPTM5	high
cg14291900	NR2C2AP	−0.6258545	3.30$\;\times \;10^{-6}$	SLC7A7	high
cg20640433	PALM2	−0.6257442	3.32$\;\times \;10^{-6}$	LAMA2	high
cg18411043	P2RY6	0.62560017	3.34$\;\times \;10^{-6}$	LAPTM5	high
cg24769499	FGF6	0.62560159	3.34$\;\times \;10^{-6}$	TMEM37	high
cg00295382	UBE4A	−0.6253707	3.37$\;\times \;10^{-6}$	MYCL	high
cg04499514	FBLIM1	−0.6251549	3.41$\;\times \;10^{-6}$	C3AR1	high
cg00777079	RFWD3	−0.6250366	3.43$\;\times \;10^{-6}$	SERPINF1	high
cg18411043	CTSB	0.62483558	3.46$\;\times \;10^{-6}$	LAPTM5	high
cg16713274	NXPH2	−0.624851	3.46$\;\times \;10^{-6}$	COL18A1	high
cg18411043	INCENP	−0.6247948	3.47$\;\times \;10^{-6}$	LAPTM5	high
cg14291900	CDK6	−0.624404	3.53$\;\times \;10^{-6}$	SLC7A7	high
cg02957057	DEFB125	0.62421976	3.56$\;\times \;10^{-6}$	NID1	high
cg18411043	CSF1R	0.62400537	3.60$\;\times \;10^{-6}$	LAPTM5	high
cg18411043	TIGD3	−0.6238521	3.62$\;\times \;10^{-6}$	LAPTM5	high
cg23986671	ATP6V0D2	−0.6237079	3.65$\;\times \;10^{-6}$	ADAMTS5	high
cg20640433	RIT1	0.62363135	3.66$\;\times \;10^{-6}$	LAMA2	high
cg14082886	SLCO1A2	0.62356807	3.67$\;\times \;10^{-6}$	CD44	high
cg18411043	ALOX5	0.62349813	3.68$\;\times \;10^{-6}$	LAPTM5	high
cg18411043	MSI1	−0.6234219	3.69$\;\times \;10^{-6}$	LAPTM5	high
cg23986671	DBH	−0.6231996	3.73$\;\times \;10^{-6}$	ADAMTS5	high
cg00295382	NDUFB2	0.62314436	3.74$\;\times \;10^{-6}$	MYCL	high
cg20640433	EVC2	−0.6228847	3.79$\;\times \;10^{-6}$	LAMA2	high
cg17599241	HAVCR2	0.6225838	3.84$\;\times \;10^{-6}$	VCAN-AS1, VCAN	high
cg18411043	RUNX2	0.62236407	3.88$\;\times \;10^{-6}$	LAPTM5	high
cg13765206	TCHH	−0.6221834	3.91$\;\times \;10^{-6}$	EMILIN2	high
cg07947930	PDCD1LG2	0.62213951	3.92$\;\times \;10^{-6}$	PRELP	high
cg18411043	POLD3	−0.6218415	3.97$\;\times \;10^{-6}$	LAPTM5	high
cg04499514	MFSD5	−0.621664	4.01$\;\times \;10^{-6}$	C3AR1	high
cg18411043	MPP1	0.62163839	4.01$\;\times \;10^{-6}$	LAPTM5	high
cg18411043	HRH2	0.62163838	4.01$\;\times \;10^{-6}$	LAPTM5	high
cg18411043	TOP2A	−0.621607	4.02$\;\times \;10^{-6}$	LAPTM5	high
cg18411043	IRAK3	0.62139951	4.06$\;\times \;10^{-6}$	LAPTM5	high
cg18397405	GPC2	−0.6210758	4.12$\;\times \;10^{-6}$	GPC6	high
cg18411043	OAF	0.62104659	4.12$\;\times \;10^{-6}$	LAPTM5	high
cg04499514	SPRY4	−0.6209967	4.13$\;\times \;10^{-6}$	C3AR1	high
cg18411043	C1S	0.6209673	4.14$\;\times \;10^{-6}$	LAPTM5	high
cg02957057	ALDH7A1	−0.6209514	4.14$\;\times \;10^{-6}$	NID1	high
cg14291900	CNOT3	−0.6209149	4.15$\;\times \;10^{-6}$	SLC7A7	high
cg02957057	TXK	−0.6207198	4.18$\;\times \;10^{-6}$	NID1	high
cg07436701	IGF1	−0.6206649	4.19$\;\times \;10^{-6}$	MMRN2, SNCG	high
cg18411043	KIF18B	−0.6205685	4.21$\;\times \;10^{-6}$	LAPTM5	high
cg18411043	ARHGAP30	0.6203351	4.26$\;\times \;10^{-6}$	LAPTM5	high
cg18411043	AIF1	0.62025359	4.27$\;\times \;10^{-6}$	LAPTM5	high
cg00295382	TGM2	0.62016995	4.29$\;\times \;10^{-6}$	MYCL	high
cg14291900	SLC26A9	0.62000399	4.32$\;\times \;10^{-6}$	SLC7A7	high
cg23986671	TMEM52	−0.6197762	4.37$\;\times \;10^{-6}$	ADAMTS5	high
cg18411043	LHFPL2	0.61974103	4.38$\;\times \;10^{-6}$	LAPTM5	high
cg14291900	SLC1A7	0.6196898	4.39$\;\times \;10^{-6}$	SLC7A7	high
cg04499514	DUSP6	−0.619504	4.42$\;\times \;10^{-6}$	C3AR1	high
cg11702456	APOBEC3F	−0.6195267	4.42$\;\times \;10^{-6}$	SP100	high
cg22568423	TGFB1	0.61936567	4.45$\;\times \;10^{-6}$	MYO1F	high
cg14082886	ADAM22	0.61922422	4.48$\;\times \;10^{-6}$	CD44	high
cg11827097	TAGLN2	−0.6185018	4.63$\;\times \;10^{-6}$	SP100	high
cg02957057	CSRP1	−0.6184356	4.64$\;\times \;10^{-6}$	NID1	high
cg21218883	HSPBP1	−0.618324	4.67$\;\times \;10^{-6}$	PRKCE	high
cg00295382	HGFAC	−0.618239	4.69$\;\times \;10^{-6}$	MYCL	high
cg14291900	GRWD1	−0.6179975	4.74$\;\times \;10^{-6}$	SLC7A7	high
cg18411043	CMKLR1	0.61781132	4.78$\;\times \;10^{-6}$	LAPTM5	high
cg18411043	CYTH4	0.61755945	4.83$\;\times \;10^{-6}$	LAPTM5	high
cg02957057	ACADS	−0.6173054	4.89$\;\times \;10^{-6}$	NID1	high
cg18411043	FANCI	−0.6170511	4.95$\;\times \;10^{-6}$	LAPTM5	high
cg20640433	LGALS8	−0.616979	4.96$\;\times \;10^{-6}$	LAMA2	high
cg04499514	CD276	−0.6168248	5.00$\;\times \;10^{-6}$	C3AR1	high
cg18411043	TNFSF10	0.61660766	5.05$\;\times \;10^{-6}$	LAPTM5	high
cg18411043	VENTX	0.61649043	5.07$\;\times \;10^{-6}$	LAPTM5	high
cg04499514	CASC3	0.61596213	5.20$\;\times \;10^{-6}$	C3AR1	high
cg09777237	TGFB1	0.61588628	5.21$\;\times \;10^{-6}$	ELN	high
cg18411043	SIGLEC10	0.61564503	5.27$\;\times \;10^{-6}$	LAPTM5	high
cg14291900	FAM124A	0.61534899	5.34$\;\times \;10^{-6}$	SLC7A7	high
cg11702456	APOBEC3C	−0.6153217	5.35$\;\times \;10^{-6}$	SP100	high
cg20640433	KHNYN	−0.6151494	5.39$\;\times \;10^{-6}$	LAMA2	high
cg14291900	RAB40B	0.61510576	5.40$\;\times \;10^{-6}$	SLC7A7	high
cg04499514	TAGLN2	−0.6149763	5.43$\;\times \;10^{-6}$	C3AR1	high
cg18411043	RAC3	−0.6149957	5.43$\;\times \;10^{-6}$	LAPTM5	high
cg14082886	EMP1	−0.6149246	5.44$\;\times \;10^{-6}$	CD44	high
cg14291900	MRVI1	0.61493337	5.44$\;\times \;10^{-6}$	SLC7A7	high
cg14082886	TAGLN2	−0.614913	5.45$\;\times \;10^{-6}$	CD44	high
cg18411043	FGD2	0.61480269	5.47$\;\times \;10^{-6}$	LAPTM5	high
cg18411043	DSE	0.6147844	5.48$\;\times \;10^{-6}$	LAPTM5	high
cg23986671	UBP1	−0.6147229	5.49$\;\times \;10^{-6}$	ADAMTS5	high
cg23986671	XKR5	−0.6145796	5.53$\;\times \;10^{-6}$	ADAMTS5	high
cg18411043	POLD4	0.61428878	5.60$\;\times \;10^{-6}$	LAPTM5	high
cg18411043	FMNL1	0.61431112	5.60$\;\times \;10^{-6}$	LAPTM5	high
cg04499514	SPAG9	0.61418162	5.63$\;\times \;10^{-6}$	C3AR1	high
cg18411043	EZH2	−0.6141715	5.63$\;\times \;10^{-6}$	LAPTM5	high
cg14291900	EFHD1	0.61415875	5.63$\;\times \;10^{-6}$	SLC7A7	high
cg18411043	TPX2	−0.6140728	5.66$\;\times \;10^{-6}$	LAPTM5	high
cg11702456	EFEMP2	−0.6138811	5.70$\;\times \;10^{-6}$	SP100	high
cg04499514	APBA1	0.61375515	5.74$\;\times \;10^{-6}$	C3AR1	high
cg01930947	DNM3	0.61362978	5.77$\;\times \;10^{-6}$	C1orf111	high
cg14082886	DAAM2	0.61357396	5.78$\;\times \;10^{-6}$	CD44	high
cg04499514	SDF2L1	−0.6132695	5.86$\;\times \;10^{-6}$	C3AR1	high
cg02957057	ACSF2	−0.6130867	5.91$\;\times \;10^{-6}$	NID1	high
cg18411043	TMEM97	−0.6127385	6.00$\;\times \;10^{-6}$	LAPTM5	high
cg18411043	CDC25A	−0.6126424	6.03$\;\times \;10^{-6}$	LAPTM5	high
cg18411043	GIMAP7	0.61247867	6.07$\;\times \;10^{-6}$	LAPTM5	high
cg14291900	ZNF45	−0.6125104	6.07$\;\times \;10^{-6}$	SLC7A7	high
cg02957057	SH2D4A	−0.6123811	6.10$\;\times \;10^{-6}$	NID1	high
cg04499514	ATP1A4	0.61222663	6.14$\;\times \;10^{-6}$	C3AR1	high
cg18411043	KIF2C	−0.6121091	6.17$\;\times \;10^{-6}$	LAPTM5	high
cg18411043	SLC20A1	0.61194867	6.22$\;\times \;10^{-6}$	LAPTM5	high
cg20640433	MMACHC	−0.6119354	6.22$\;\times \;10^{-6}$	LAMA2	high
cg18411043	ECM1	0.61187776	6.24$\;\times \;10^{-6}$	LAPTM5	high
cg00295382	C5orf51	−0.6118449	6.25$\;\times \;10^{-6}$	MYCL	high
cg18411043	CMTM7	0.61176944	6.27$\;\times \;10^{-6}$	LAPTM5	high
cg04499514	EHD4	−0.611663	6.30$\;\times \;10^{-6}$	C3AR1	high
cg18411043	CRISPLD2	0.61159373	6.32$\;\times \;10^{-6}$	LAPTM5	high
cg00295382	ATF7IP	−0.6113318	6.39$\;\times \;10^{-6}$	MYCL	high
cg07436701	CD163	−0.6113281	6.39$\;\times \;10^{-6}$	MMRN2, SNCG	high
cg21398469	TGFB1	0.61126793	6.41$\;\times \;10^{-6}$	CCNG2	high
cg07436701	CD244	−0.6112525	6.41$\;\times \;10^{-6}$	MMRN2, SNCG	high
cg14291900	ABCG1	0.61123795	6.42$\;\times \;10^{-6}$	SLC7A7	high
cg14291900	ZNF761	−0.6110213	6.48$\;\times \;10^{-6}$	SLC7A7	high
cg18411043	HHEX	0.61088519	6.52$\;\times \;10^{-6}$	LAPTM5	high
cg22595235	CTLA4	0.61078204	6.55$\;\times \;10^{-6}$	SUMF1, LRRN1	high
cg24769499	IL22	0.61065046	6.59$\;\times \;10^{-6}$	TMEM37	high
cg18411043	MNDA	0.61034153	6.68$\;\times \;10^{-6}$	LAPTM5	high
cg18411043	FAH	0.61025131	6.71$\;\times \;10^{-6}$	LAPTM5	high
cg11702456	SP100	−0.6102275	6.71$\;\times \;10^{-6}$	SP100	high
cg23986671	DUOXA1	−0.6102538	6.71$\;\times \;10^{-6}$	ADAMTS5	high
cg00295382	PANK3	−0.6101989	6.72$\;\times \;10^{-6}$	MYCL	high
cg18411043	CLEC10A	0.61013964	6.74$\;\times \;10^{-6}$	LAPTM5	high
cg18411043	TRAF3IP3	0.61007507	6.76$\;\times \;10^{-6}$	LAPTM5	high
cg13765206	CAPN8	−0.6097062	6.87$\;\times \;10^{-6}$	EMILIN2	high
cg14291900	PAQR8	0.60959693	6.90$\;\times \;10^{-6}$	SLC7A7	high
cg02957057	SDC4	−0.609594	6.90$\;\times \;10^{-6}$	NID1	high
cg20640433	ISPD	−0.6095796	6.91$\;\times \;10^{-6}$	LAMA2	high
cg08064683	CCR4	0.60946563	6.94$\;\times \;10^{-6}$	FAT1	high
cg04499514	AP2S1	−0.6092851	7.00$\;\times \;10^{-6}$	C3AR1	high
cg04499514	ITPRIP	−0.60917	7.03$\;\times \;10^{-6}$	C3AR1	high
cg04499514	ADHFE1	0.60916978	7.03$\;\times \;10^{-6}$	C3AR1	high
cg00295382	ARPC1B	0.60912481	7.05$\;\times \;10^{-6}$	MYCL	high
cg18411043	ZNF90	−0.6090191	7.08$\;\times \;10^{-6}$	LAPTM5	high
cg00295382	CREBZF	−0.6089524	7.10$\;\times \;10^{-6}$	MYCL	high
cg14291900	DPEP2	0.60894355	7.10$\;\times \;10^{-6}$	SLC7A7	high
cg02957057	CCDC163P	−0.608834	7.14$\;\times \;10^{-6}$	NID1	high
cg04499514	AKAP1	0.60866034	7.19$\;\times \;10^{-6}$	C3AR1	high
cg20640433	SLC2A10	−0.6085969	7.21$\;\times \;10^{-6}$	LAMA2	high
cg14291900	TPD52L1	0.60852744	7.24$\;\times \;10^{-6}$	SLC7A7	high
cg11827097	PRMT2	−0.6081478	7.36$\;\times \;10^{-6}$	SP100	high
cg23986671	PRPH2	−0.6081063	7.37$\;\times \;10^{-6}$	ADAMTS5	high
cg18397405	ITGB1	0.60795654	7.42$\;\times \;10^{-6}$	GPC6	high
cg02957057	RRP12	0.60790296	7.44$\;\times \;10^{-6}$	NID1	high
cg18411043	ADAP2	0.60781942	7.46$\;\times \;10^{-6}$	LAPTM5	high
cg18411043	CCR1	0.60783567	7.46$\;\times \;10^{-6}$	LAPTM5	high
cg18411043	IL15RA	0.60780418	7.47$\;\times \;10^{-6}$	LAPTM5	high
cg11702456	CMTM3	−0.6077214	7.50$\;\times \;10^{-6}$	SP100	high
cg04499514	FBXW12	0.60765521	7.52$\;\times \;10^{-6}$	C3AR1	high
cg14291900	ADRBK2	0.60758355	7.54$\;\times \;10^{-6}$	SLC7A7	high
cg18411043	WDR34	−0.6072402	7.66$\;\times \;10^{-6}$	LAPTM5	high
cg18411043	LAIR1	0.60714771	7.69$\;\times \;10^{-6}$	LAPTM5	high
cg00295382	ZBTB44	−0.6070874	7.71$\;\times \;10^{-6}$	MYCL	high
cg13765206	NRAP	−0.6067666	7.82$\;\times \;10^{-6}$	EMILIN2	high
cg14291900	SLCO1A2	0.60635008	7.96$\;\times \;10^{-6}$	SLC7A7	high
cg16713274	OLFM3	−0.6062793	7.99$\;\times \;10^{-6}$	COL18A1	high
cg00295382	FAM166A	−0.6061765	8.02$\;\times \;10^{-6}$	MYCL	high
cg02957057	RAB36	−0.6061395	8.03$\;\times \;10^{-6}$	NID1	high
cg14291900	TMEM86A	0.60607985	8.05$\;\times \;10^{-6}$	SLC7A7	high
cg14291900	EVI2A	0.60605156	8.06$\;\times \;10^{-6}$	SLC7A7	high
cg18411043	CTSZ	0.60597807	8.09$\;\times \;10^{-6}$	LAPTM5	high
cg13765206	NCR3	−0.6057964	8.16$\;\times \;10^{-6}$	EMILIN2	high
cg13765206	KRTAP5-9	−0.605737	8.18$\;\times \;10^{-6}$	EMILIN2	high
cg18411043	HES5	−0.6056663	8.20$\;\times \;10^{-6}$	LAPTM5	high
cg11702456	ARSI	−0.6056573	8.20$\;\times \;10^{-6}$	SP100	high
cg18411043	MFSD1	0.60562112	$8.22\;\times \;10^{-6}$	LAPTM5	high
cg00295382	ZG16	−0.6055812	$8.23\;\times \;10^{-6}$	MYCL	high
cg20640433	DPEP3	−0.6054516	$8.28\;\times \;10^{-6}$	LAMA2	high
cg18411043	MAP2	−0.6053901	$8.30\;\times \;10^{-6}$	LAPTM5	high
cg18411043	ADAMTS14	0.60538276	$8.30\;\times \;10^{-6}$	LAPTM5	high
cg04499514	KDELR1	−0.605255	$8.35\;\times \;10^{-6}$	C3AR1	high
cg04499514	RALGPS1	0.60522199	$8.36\;\times \;10^{-6}$	C3AR1	high
cg18411043	BRIP1	−0.6052277	$8.36\;\times \;10^{-6}$	LAPTM5	high
cg14291900	DLEU7	0.60520069	$8.37\;\times \;10^{-6}$	SLC7A7	high
cg18411043	RNF149	0.60510698	$8.40\;\times \;10^{-6}$	LAPTM5	high
cg18411043	LEPROT	0.60482116	$8.51\;\times \;10^{-6}$	LAPTM5	high
cg18411043	GIMAP4	0.60467954	$8.56\;\times \;10^{-6}$	LAPTM5	high
cg00295382	RGS19	0.60458767	$8.60\;\times \;10^{-6}$	MYCL	high
cg18411043	IL10RA	0.60450736	$8.63\;\times \;10^{-6}$	LAPTM5	high
cg18411043	SLCO2B1	0.60445283	$8.65\;\times \;10^{-6}$	LAPTM5	high
cg00295382	TTC38	0.60437946	$8.67\;\times \;10^{-6}$	MYCL	high
cg14291900	PTBP1	−0.6043814	$8.67\;\times \;10^{-6}$	SLC7A7	high
cg18411043	IL16	0.60434727	$8.69\;\times \;10^{-6}$	LAPTM5	high
cg04499514	PPIB	−0.6040378	$8.80\;\times \;10^{-6}$	C3AR1	high
cg18411043	MAPKAPK2	0.60396534	$8.83\;\times \;10^{-6}$	LAPTM5	high
cg04499514	TGFBI	−0.6038334	$8.88\;\times \;10^{-6}$	C3AR1	high
cg04499514	IGFBP2	−0.6037505	$8.91\;\times \;10^{-6}$	C3AR1	high
cg11702456	SLC2A4	0.60343179	$9.04\;\times \;10^{-6}$	SP100	high
cg04499514	IKBIP	−0.603409	$9.05\;\times \;10^{-6}$	C3AR1	high
cg04499514	ETV5	−0.603302	$9.09\;\times \;10^{-6}$	C3AR1	high
cg12613839	PDCD1LG2	0.60313469	$9.15\;\times \;10^{-6}$	ADAMTS2	high
cg04499514	KIAA1324L	0.60307321	$9.18\;\times \;10^{-6}$	C3AR1	high
cg18411043	FMN1	0.60306718	$9.18\;\times \;10^{-6}$	LAPTM5	high
cg18411043	SH3TC1	0.6029736	$9.22\;\times \;10^{-6}$	LAPTM5	high
cg02957057	LEKR1	−0.6029529	$9.23\;\times \;10^{-6}$	NID1	high
cg18411043	GRB2	0.60266775	$9.34\;\times \;10^{-6}$	LAPTM5	high
cg04499514	PSD2	0.60262221	$9.36\;\times \;10^{-6}$	C3AR1	high
cg11702456	CASP8	−0.6025684	$9.38\;\times \;10^{-6}$	SP100	high
cg04499514	IFNGR2	−0.6025283	$9.40\;\times \;10^{-6}$	C3AR1	high
cg14082886	DCAF8	0.60237965	$9.46\;\times \;10^{-6}$	CD44	high
cg02957057	C10orf107	−0.6023697	$9.46\;\times \;10^{-6}$	NID1	high
cg04499514	CKAP4	-0.6023102	$9.49\;\times \;10^{-6}$	C3AR1	high
cg02957057	RTP2	0.60230352	$9.49\;\times \;10^{-6}$	NID1	high
cg18411043	RFC5	−0.6022106	$9.53\;\times \;10^{-6}$	LAPTM5	high
cg11827097	TSEN34	−0.6020259	$9.60\;\times \;10^{-6}$	SP100	high
cg18411043	YWHAZ	0.60194076	$9.64\;\times \;10^{-6}$	LAPTM5	high
cg02957057	TMIE	−0.6019364	$9.64\;\times \;10^{-6}$	NID1	high
cg02957057	GLIS3	−0.6017402	$9.72\;\times \;10^{-6}$	NID1	high
cg00295382	TRO	−0.60165	$9.76\;\times \;10^{-6}$	MYCL	high
cg20640433	FAM19A1	−0.6014471	$9.84\;\times \;10^{-6}$	LAMA2	high
cg18411043	LCORL	−0.6013862	$9.87\;\times \;10^{-6}$	LAPTM5	high
cg20640433	PAOX	−0.6011955	$9.95\;\times \;10^{-6}$	LAMA2	high
cg14291900	SAE1	−0.6011065	$9.99\;\times \;10^{-6}$	SLC7A7	high
cg18411043	DENND1C	0.60107737	$1.00\;\times \;10^{-5}$	LAPTM5	high
cg00295382	ZNF510	−0.6009576	1.01$\;\times \;10^{-5}$	MYCL	high
cg18411043	MED24	−0.6008814	1.01$\;\times \;10^{-5}$	LAPTM5	high
cg14082886	ATP8A1	0.60058342	1.02$\;\times \;10^{-5}$	CD44	high
cg18411043	RAD54L	−0.600678	1.02$\;\times \;10^{-5}$	LAPTM5	high
cg18411043	SP4	−0.6005993	1.02$\;\times \;10^{-5}$	LAPTM5	high
cg04499514	TIMP1	−0.6001084	1.04$\;\times \;10^{-5}$	C3AR1	high
cg14291900	RASGEF1B	0.60012159	1.04$\;\times \;10^{-5}$	SLC7A7	high
cg02957057	ZDHHC1	−0.6000678	1.04$\;\times \;10^{-5}$	NID1	high
cg02957057	HRASLS5	−0.6000243	1.05$\;\times \;10^{-5}$	NID1	high
cg07436701	CD96	−0.5993043	1.08$\;\times \;10^{-5}$	MMRN2, SNCG	high
cg07436701	CCR4	−0.5972957	1.18$\;\times \;10^{-5}$	MMRN2, SNCG	high
cg18397405	ITGA4	0.59418147	1.34$\;\times \;10^{-5}$	GPC6	high
cg03677069	CD74	0.59295197	$1.41\;\times \;10^{-5}$	MMRN2, SNCG	high
cg07436701	GPR65	−0.5925407	$1.43\;\times \;10^{-5}$	MMRN2, SNCG	high
cg18397405	CDC34	−0.590695	$1.55\;\times \;10^{-5}$	GPC6	high
cg07436701	FLT3	−0.5827163	$2.15\;\times \;10^{-5}$	MMRN2, SNCG	high
cg07436701	GPC2	0.5754121	$2.87\;\times \;10^{-5}$	MMRN2, SNCG	high
cg07436701	E2F2	0.5728996	$3.17\;\times \;10^{-5}$	MMRN2, SNCG	high
cg07436701	CD14	−0.568245	$3.80\;\times \;10^{-5}$	MMRN2, SNCG	high
cg18397405	EZH2	−0.5582639	$5.54\;\times \;10^{-5}$	GPC6	high
cg18397405	CDKN1B	−0.5581881	$5.56\;\times \;10^{-5}$	GPC6	high
cg07436701	CDC34	0.55769852	$5.66\;\times \;10^{-5}$	MMRN2, SNCG	high
cg07436701	CD68	−0.5556475	$6.11\;\times \;10^{-5}$	MMRN2, SNCG	high
cg26350754	EMILIN2	−0.554466	$6.38\;\times \;10^{-5}$	HLA-DPA1, HLA-DPB1	high
cg14082886	MRC2	−0.5539332	$6.51\;\times \;10^{-5}$	CD44	high
cg18397405	E2F2	−0.5518593	$7.02\;\times \;10^{-5}$	GPC6	high
cg18397405	FLT3	0.54987539	$7.55\;\times \;10^{-5}$	GPC6	high
cg10949632	GPC6	0.54286496	$9.70\;\times \;10^{-5}$	GPC6	high
cg03677069	GPR65	0.54188941	0.00010045	MMRN2, SNCG	high
cg14082886	FGFR2	0.5340032	0.00013232	CD44	high
cg16713274	GPC6	−0.5317779	0.00014285	COL18A1, LL21NC02-21A1.1	high
cg03677069	CD163	0.52832557	0.00016069	MMRN2, SNCG	high
cg21012874	CD74	0.52760147	0.00016467	MMRN2, SNCG	high
cg09552892	CD74	0.52237375	0.00019625	MMRN2, SNCG	high
cg04499514	EZH1	0.5201497	0.00021127	C3AR1	high
cg07436701	EZH2	0.51876207	0.00022116	MMRN2, SNCG	high
cg14082886	CD63	−0.5173898	0.00023136	CD44	high
cg04098585	EMILIN2	−0.5123153	0.00027286	CD28	high
cg07436701	GZMA	−0.5121674	0.00027417	MMRN2, SNCG	high
cg03677069	ITGB2	0.5110316	0.00028438	MMRN2, SNCG	high
cg07436701	CCL5	−0.5105968	0.00028837	MMRN2, SNCG	high
cg03677069	E2F2	−0.5095174	0.00029852	MMRN2, SNCG	high
cg03677069	CD14	0.50607605	0.00033306	MMRN2, SNCG	high
cg04499514	FGFR1	−0.5048488	0.00034622	C3AR1	high
cg07436701	GRN	−0.5045134	0.0003499	MMRN2, SNCG	high
cg18397405	CD63	0.50108656	0.00038956	GPC6	high
